# Cue integration during sentence comprehension: Electrophysiological evidence from ellipsis

**DOI:** 10.1371/journal.pone.0206616

**Published:** 2018-11-29

**Authors:** Andrea E. Martin

**Affiliations:** 1 Department of Psychology, School of Philosophy, Psychology and Language Sciences, University of Edinburgh, Edinburgh, United Kingdom; 2 Max-Planck Institute for Psycholinguistics, Nijmegen, The Netherlands; National Institutes of Health, UNITED STATES

## Abstract

Language processing requires us to integrate incoming linguistic representations with representations of past input, often across intervening words and phrases. This computational situation has been argued to require retrieval of the appropriate representations from memory via a set of features or representations serving as retrieval cues. However, even within in a cue-based retrieval account of language comprehension, both the structure of retrieval cues and the particular computation that underlies direct-access retrieval are still underspecified. Evidence from two event-related brain potential (ERP) experiments that show cue-based interference from different types of linguistic representations during ellipsis comprehension are consistent with an architecture wherein different cue types are integrated, and where the interaction of cue with the recent contents of memory determines processing outcome, including expression of the interference effect in ERP componentry. I conclude that retrieval likely includes a computation where cues are integrated with the contents of memory via a linear weighting scheme, and I propose vector addition as a candidate formalization of this computation. I attempt to account for these effects and other related phenomena within a broader cue-based framework of language processing.

## Introduction

Language comprehension requires the generation of hierarchical linguistic representations across multiple timescales. The appropriate representations within a hierarchical sentence structure must be interpreted together, even though they are often separated from each other in time and space. For example:

(1) The child who loved claiming that the moon belonged to him smiled.

Though in (1), the noun phrase *the moon* is closer to the verb, it is the noun phrase *the child who loved claiming that the moon belonged to him* that must be integrated with *smiled* as the subject of that verb phrase. This representational configuration means that phrasal computations must span over intervening words and phrases, such that certain representations, but not others, are combined and interpreted together. This computational challenge can become even more complex due a powerful compression algorithm in human language–ellipsis, where information (words and phrases) that is already given in the discourse can be omitted (i.e., not pronounced) but still understood, as in (2):

(2) The child searched for the moon in the night sky, and after some cajoling and whining, his father did too.

In (2), the phrase *did too* stands in for the phrase *searched for the moon in the night sky*. The information in the latter phrase can be compressed into the former, and then recovered and interpreted in a new position in the sentence with a new referent as its subject. But how is the relationship between the two forms, between the antecedent and the ellipsis, established in the first place? How are other intervening words, phrases, or other meanings, excluded as antecedents for the ellipsis? In other words, what information links the antecedent to the ellipsis site, or cues it in memory?

In this paper, I present data from two event-related brain potential experiments that show interference as function of the relation between recently processed linguistic information, and the features of a critical phrase that is intended to be elliptical. I argue that these effects are consistent with a computational architecture wherein different levels of linguistic representation in the current input or processing moment are combined via a linear weighting scheme and then integrated with the contents of memory using vector addition in order to elicit an antecedent from memory.

Ellipsis presents a complex computational challenge to the parser. Many linguistic dependencies, for example, subject-verb dependencies, as in (1), make use of explicit structural information between the verb and noun, for example, between *smiled* and *the child* in (1), to relate the appropriate constituents to each other and to exclude inappropriate representations like *the moon*. Other relevant types of information, such as the animate subject requirement of *smiled*, person and number features (regardless of morphological syncretism in English) presumably further cue the relationship between *smiled* and *the child*. However, under ellipsis, e.g., in (2), the source of the information leading to the right meaning for *did too* is less well-understood. This paper seeks to understand what information that leads to the recovery of elided meaning from memory, and to infer how that recovery might occur by manipulating both information in memory and the status of retrieval cues.

In (1) and (2), the right representations must be recovered from memory in order for language comprehension to occur. Early insight in linguistics and psycholinguistics has invoked a pivotal role for memory in online language processing (e.g., [1]). In the last two decades, theories about the computational infrastructure of memory for language processing have grown more articulated, namely, as a basic recognition memory architecture for processing linguistic representations, or the *cue-based retrieval* framework [2–15]. Cue-based retrieval aims to extend the computational architecture of human recognition memory and its mechanistic principles [11, 16, 17] to language processing contexts; the focus of the framework thus far has been retrieval from memory during long-distance dependency formation, as in the examples above. Cue-based retrieval does not specify any computational difference between word recognition and retrieval of a displaced or distal constituent, though there are likely important differences, which could be expressed either in terms of threshold of activation required or in terms of the structured representations that are activated. In the framework, the main determinant of whether a representation is successfully recovered from memory is *interference*, which arises as a function of retrieval cues matching more than one item in memory, such that the presence of similar items in memory lowers the likelihood of retrieval for a given target since retrieval cues are no longer diagnostic to a single, unique item in memory. I will briefly sketch the computational architecture that gives rise to cue-based retrieval interference as the primary determinant of processing difficulty.

In the cue-based framework, retrieval is required whenever a representation that is no longer in the current focus of attention is needed for interpretation to occur [11]. Experiments measuring processing speed during retrieval have used subject-verb dependencies [9, 13], pronouns [2], and ellipsis [6–8]. These findings, along with others from the memory literature, suggest that retrieval occurs via contact of retrieval cues with representations in memory, and is a *direct-access* operation. The main argument for direct-access retrieval (and against engagement of a serial search of memory, as recruited to recover the temporal order information needed in judgements of recency and n-back tasks; see [17, 18] is that retrieval speed is constant in both list-learning and sentence contexts: recognition judgments on items from outside of the focus of attention take, on average, ~250msec longer compared to responses to items in the focus of attention. Under a search, retrieval speed would vary as a function of set size, distance, or serial position of a target. It simply does not appear to do so [11, 16].

Direct-access retrieval has significant implications for the computational architecture of language processing, as well as for that of semantic memory representations more generally. It strongly implies that memory representations are organised and found by virtue of their content, *i*.*e*., are *content-addressable*, such that retrieval can occur without a search through memory [4, 6–9, 16]. In such an architecture, the content-addressability of representations is a powerful first principle that determines processing outcome—information at the moment of retrieval, or retrieval cues, must make contact with other representations (or their features) in memory to elicit the target representation [11]. But, if other “non-target” representations match or partially match the cues, the likelihood of successful retrieval of the target representation necessarily decreases. Thus, this powerful first principle of content-addressability leads to another: susceptibility to interference from similar items in memory. Distinctiveness can also be disruptive to retrieval, but the true spectrum or continuum of interference effects as a function of similarity or distinctiveness is still unknown [19–22].

Under this architecture, the extent to which ‘unintended’ memory representations with content-overlap interfere with retrieval naturally emerges as the primary determinant of retrieval difficulty and forgetting [11, 23, 24], and therefore of sentence processing difficulty during dependency resolution [4, 12, 13, 14, 19, 20, 25–30]. This decrease in likelihood of successful retrieval is known as *cue-based retrieval interference*. Cue-based interference is a function of cue overload, which is also described or formalized as *cue diagnosticity*–the match between cues and the target divided by the match between cues with other items in memory [5, 19, 23].

### Cue-based interference effects and their representational sources

The cue-based architecture naturally prompts the question as to what information serves as retrieval cues during dependency resolution. Minimally, some basic representation types are obvious–in subject-verb dependencies, information on the displaced verb must somehow lead to retrieval of the subject, whereas a pronoun signals its antecedent in some languages via gender agreement, and verb phrase ellipsis, in English, its antecedent via voice and other features. Representation types implicated as retrieval cues, and thus argued to create interference, span a range in the extant literature. For example, semantic features on noun phrases, which also impact their referential status, create an interference pattern such that proper or occupation nouns tend to interfere with each other more than a proper name or pronoun does [25, 26]. The structural relationship between dependent elements is clearly of central importance, but the evidence is mixed as to how fungible structural constraints can be [14, 19, 20, 31–37], and it still unclear how structural relations are computed and passed forward incrementally during processing—nonetheless structural cues are no doubt crucial. Animacy is another dimension along which retrieval interference appears to accrue–animate nouns in memory, but not inanimate ones, interfere with retrieval when the cues on a verb demand an animate subject [12, 14, 38]. Effects of biological and grammatical gender on reflexive anaphor resolution can also be seen as a form of interference effect [21, 22]. Furthermore, the large literature on agreement attraction can also be interpreted as a series of interference effects, wherein morphosyntactic features interfere with each other during both in production and comprehension [30, 39, 40]. Similarly, so-called grammatical illusions, where ungrammatical structures or semantically anomalous representations go temporarily unnoticed as such, might arise from a degree of cue-match that obscures or masks the underlying representational insufficiency [15, 37, 41, 42]. There is also behavioural evidence that agreement features can be projected forward and affect perception of dependent morphemes [43].

Other types of information that are either nominally present or given in the processing context, such as prominence, focus, or givenness, might also serve as features on the target in memory or as retrieval cues. One way to discover the role of information that is not explicit or overtly coded, is to manipulate not only aspects of the overt retrieval cue, but also of the recent contents of memory to observe what sorts of representations are disruptive to retrieval and to subsequent interpretation or processing. If interference effects can be modulated as a function of manipulation of different kinds of information, that would suggest that cues are integrated or combined at retrieval to elicit the target.

### The current study

In order to investigate what information serves as retrieval cues, and to observe how those cues interface with memory, a linguistic construction is needed where (1) material before the dependency site can be manipulated without introducing ambiguity, and (2) overt cues at the retrieval site can be manipulated. Crossing these two factors will allow the observation of the interaction between retrieval cues and contents of memory and shed light both on cue representations and the computational mechanism by which cues probe memory. Ellipsis, where part of a representation can be omitted yet is still understood, provides a useful lens because it allows both these factors to be manipulated. Crossing cue validity of the ellipsis site (*e*.*g*., *Because Jane drank the cocktail that was served by the waiter*, Bill did/*was too), with a manipulation of the representations that were recently processed (*i*.*e*., voice features of active versus passive on a verb phrase in a relative clause that cannot be an antecedent for the ellipsis–*Because Jane drank the cocktail that the waiter served*, Bill did/*was too.) yields such a processing context. In the resulting violation paradigm, a cue is Valid or Invalid, such that it either had matching or mismatching voice to the antecedent, rendering the ellipsis grammatical or ungrammatical. Only when the voice of the cue matches the antecedent, is it successfully elicited; this means that in the case of a passive antecedent, passive voice cues elicit the antecedent while active voice cues do not, and vice versa. The attractor verb phrase occurred in a relative clause, and therefore could never be an antecedent for the ellipsis site. The voice of the attractor verb phrase was manipulated to match the antecedent and the Correct cue (Attractor Same) or to be different (Attractor Different) from the antecedent and Correct cue (see Methods below for an example and Appendix 1 for a full list of stimuli).

### Predictions

One possibility is that cue validity does not interact with recently processed representational features in memory, because those features or representations are not in a syntactically-licensed position for the ellipsis. If this is the case, then a main effect of cue validity would be observed, and it would indicate that retrieval cues are either structurally diagnostic to the antecedent, or not composed of multiple information types. On the other hand, if partial cue-match to recently processed (but syntactically illicit) representations in memory is observed, an interaction between cue validity and attractor voice is predicted. Such a pattern would indicate that multiple types of cues are integrated, but that the recent contents of memory controls how information types affect processing. In other words, if composite retrieval cues are compared with all of memory, not privileged representations or locations, then latent partial cue-match will disrupt processing. The existing literature predicts that the disruption could be driven by mean differences between all four conditions: (1) a > b: Prediction of classic similarity-based interference, such that matching voice on retrieval cue matches more than one verb phrase in memory and is thus disruptive to retrieval, (2) b > a: Consistent with [19, 20] where during computation of morphosyntactic agreement, distinctiveness of a morpheme was disruptive to retrieval even during grammatical ellipsis with a valid cue, (3) c > d: Consistent with [19, 20] in that distinctiveness is disruptive, but also when no grammatical antecedent is readily elicited, or under ungrammatical ellipsis, which was not observed by [19, 20]., (4) d > c: Classic prediction of similarity-based interference but only arises in the absence of a readily elicited grammatical antecedent.

### Event-related brain potentials as an index of cue-based interference during sentence comprehension

One way to observe processing to test the interaction predictions above is by calculating event-related brain potentials (ERPs). ERPs offer multidimensional (polarity, morphology, scalp-distribution) information about whether the brain, and by inference, cognitive processing, is differentially sensitive to linguistic or other representational manipulations [44, 45]. Furthermore, ERPs register processing on a millisecond timescale and can offer a sensitivity that behavioural paradigms sometimes cannot offer, especially when attempting to observe transient representational states over time, which may or may not affect behavioural outcomes [19, 44–46]. Event-related brain potentials can offer a nuanced view of interference effects, reflecting them even when they might not affect overt behavioural responses [19, 20]. However, the literature suggests mixed predictions in terms of the components likely to be elicited in this ellipsis paradigm [47, 48, 49]. In the aforementioned ERP literature on ellipsis-related phenomena, many variants have been tested, but differences in stimuli characteristics (e.g., syntactic category, language, syntactic licensing, plausibility and other manipulations) makes generating precise or highly-specified ERP predictions difficult. On balance, given the morphosyntactic agreement violation is the root of the Cue Validity manipulation, an amplitude modulation of the P600 component is expected, including an expression of one of the interaction patterns described above [19, 20, 27, 50].

## 1 Experiment 1

### 1.1 Methods

#### 1.1.1 Participants

Twenty- four British English monolingual native speakers (19 females) aged between 18 and 34 years (*M* = 22 years) participated in the experiment, one was excluded for poor data quality, leading to a total of twenty-three participants. An additional participant was excluded from analyses due to excessive artefacts. All participants were right-handed and were free from neurological or language disorders. None of them have participated in the pre-test that is described in the Stimuli and Experimental Design section. Ethics approval was obtained the School of Philosophy, Psychology, and Language Sciences research ethics committee (230-1617/2). Written informed consent was obtained from participants and their data were anonymized.

#### 1.1.2 Stimuli and experimental design

The experimental stimuli consisted of 156 quadruplets like (a-d) in Table 1.

**Table 1:** 

Condition	Sentence
Valid Cue, Same Voice	a) Because Jane got the meal that the takeaway sold that night, John did too, as usual.
Valid Cue, Different Voice	b) Because Jane got the meal that was sold by the takeaway that night, John did too, as usual.
Invalid Cue, Different Voice	c) Because Jane got the meal that the takeaway sold that night, John was too, as usual.
Invalid Cue, Same Voice	d) Because Jane got the meal that was sold by the takeaway that night, John was too, as usual.

These four conditions were based on a 2 (Cue Validity: Valid vs. Invalid cue) by 2 (Attractor Voice: Same vs. Different) fully-crossed design. Cue Validity was determined by whether the phrase *was too* or *did too* corresponded with the voice features of the antecedent verb phrase. In the Same Voice conditions (a & c), the antecedent and the attractor verb phrase in the relative clause had the same active or passive voice (*got the meal*–*sold (the meal)*), whereas in the Different voice conditions (b & d), the main verb was active and the verb in the relative clause was passive (*got the meal*–*was sold by*), or vice versa. The voice of the main verb phrase was counterbalanced (i.e., half the sentences were active and the other half was passive). For each item, one grammatical and one ungrammatical control sentence that did not contain a relative clause were created (“*Because Jane got the meal after a cocktail at brunch*, *Erik did too/ was too*, *as usual*.”). Additional 160 grammatical sentences that did not have ellipsis were included as fillers.

#### 1.1.3 Procedure

The 316 items were divided into six counterbalanced lists, so that each list contained one of the six (four critical and two control) conditions per item and had the same number of sentences from each condition. The sentences were pseudorandomised such that no condition appeared more than three times consecutively.

Participants silently read each sentence from a computer display, presented in the centre of the display word by word at a word duration of 300 ms (200 ms inter-word interval, sentence final word had a 600 ms word duration), except the critical words *was too/ did too* that were presented together. A fixation-cross followed each sentence, at which point participants could start the next sentence by making a button-press. Yes-No comprehension questions appeared on 25% of the trials (mean accuracy = 91.3%, *SD* = 4.6%, range = 78.2–98.5%, 16% of the responses are excluded due to time outs). After the EEG experiment, participants performed a computerised Reading Span Test [51]. Their task was to read aloud random sets of two to six unrelated sentences and to recall every final word after each set. The total number of words recalled was used as their reading span. The experiment took approximately 70 minutes.

#### 1.1.4 Electroencephalogram (EEG) recording and data pre-processing

The electroencephalogram (EEG) was recorded at a sample rate of 512 Hz and with 24-bit AD conversion using the Biosemi ActiveTwo system (BioSemi BV, Amsterdam, The Netherlands). The system was DC coupled and a digital low pass was applied approximating a low-pass filter at 100 Hz. Data was recorded from 64 EEG, 4 EOG, and 2 mastoid electrodes using the standard 10/20 system. Offline, the EEG was re-referenced to the mastoid average and filtered further (0.2–20 Hz plus 50 Hz Notch filter). Data was segmented into 1200 ms epochs (-200–1000 ms relative to critical word onset), corrected for eye-movements using the Gratton and Coles regression procedure as implemented in BrainVision Analyzer (Brain Products), baseline-corrected to 0–200 ms, automatically screened for movement- or electrode-artefacts (minimal/maximal allowed amplitude = -75/75 μV), and averaged per condition per participant. The mean proportion of artefact-free trials across conditions was 89% (*SD* = 10%), with no difference across conditions. Using a standard baseline (-100–0 ms) left visible ERP differences in an early time window despite the fact that the three words preceding the critical word were identical across conditions. Specifically, the Grammatical Attractor Same condition elicited more negative ERPs relative to the other three conditions between around 0–200 ms. To avoid this difference affecting the critical analysis in a later time window, the data were analysed using a post-stimulus baseline [52–55].

#### 1.1.5 Statistical analysis

Mean amplitude was computed for frontal and posterior channels separately, and per participant and per item, at 26 frontal electrodes (Fp1/AF3/AF7/F1/F3/F5/F7/FC1/FC3/FC5/FT7 plus right-hemisphere equivalents, and Fpz/AFz/Fz/FCz) in the time windows 600–800 ms and 800–1000 ms to cover the traditional window of the P600 component, and at 26 posterior electrodes (O1/PO3/PO7/P1/P3/P5/P7/PC1/PC3/PC5/TP7 plus right-hemisphere equivalents, and Oz/POz/Pz/PCz) in the same time windows. A linear mixed-effects model evaluated the ERP amplitude predicted by the fixed effects of Cue Validity (Valid vs. Invalid) and Voice (Same vs. Different), and by the interaction of the two. The Cue Validity and Voice factors were contrast-coded using deviation coding. The model additionally included random intercepts by participants and by items.

### 1.2 Results

**Fig 1:**
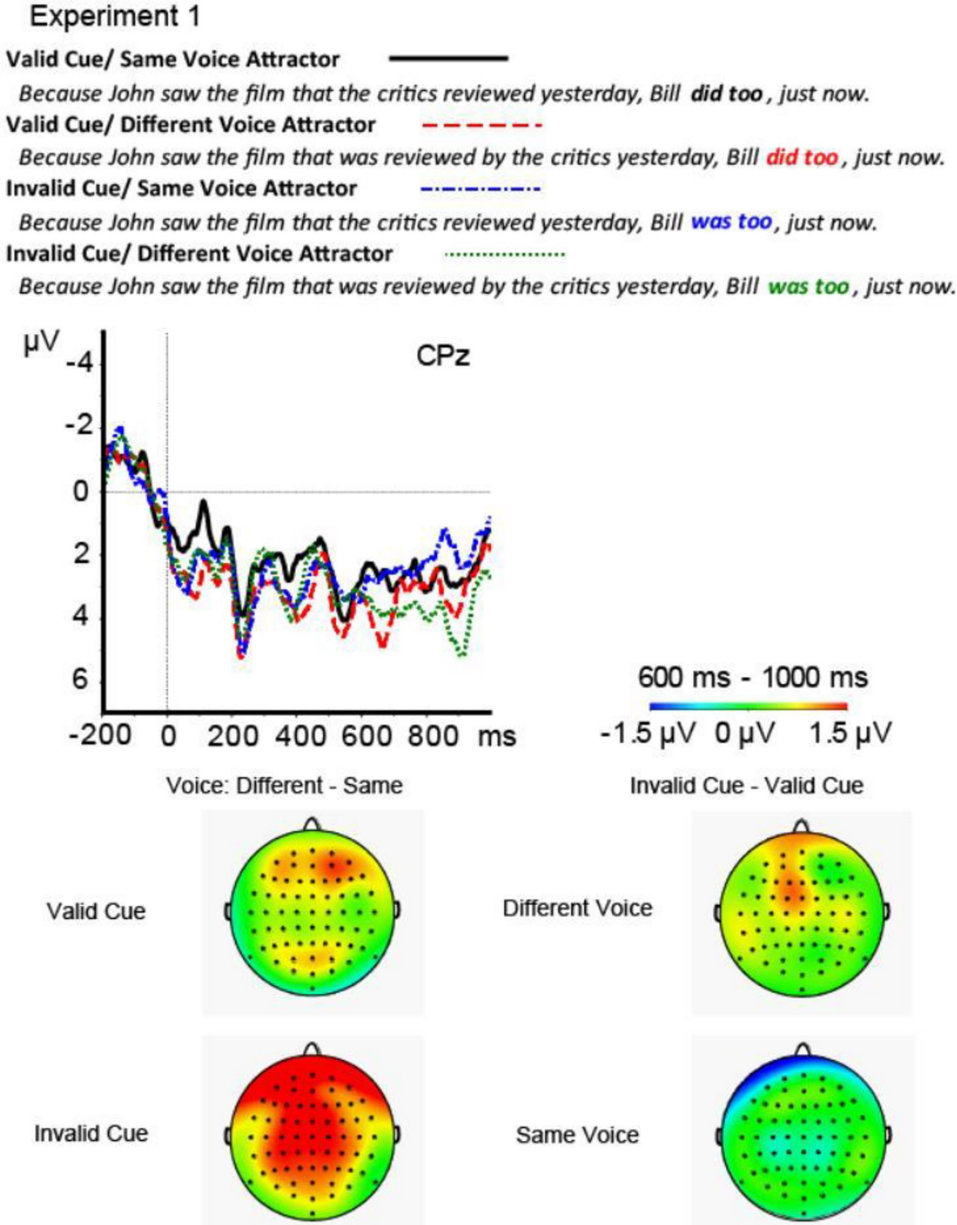
Results from Experiment 1. ERPs elicited by each condition at FCz, Pz, and the scalp distributions of the effect of Cue Validity for the Different Voice and the Same Voice conditions in the 800–1000 ms time window. The ERP waveforms on FCz are representative of the pattern across frontal electrodes while the waveforms on Pz are representative of those across posterior electrodes. Please see Appendix B for figures showing all electrodes.

Visual inspection of the ERP suggests that when the attractor had a Different Voice, Invalid Cue critical phrases elicited a larger positivity relative to Valid Cue equivalents starting from about 700 ms, the difference peaking at around 900 ms (see Fig 1). This effect was broadly distributed at frontal channels, suggesting a frontal P600 effect. In contrast, when the attractor had the Same Voice as the antecedent, Valid Cue critical phrases elicited a larger positivity than Invalid Cue equivalents. A model with random slopes of Cue Validity and Voice was also run, but it did not converge, even when the interaction term was removed.

I report the mean difference (β), standard error (*SE*) and *t*-values, where *t > |*2| indicates a significant effect. The linear mixed-effects models for both frontal and posterior channels did not show any significant effects for the 600–800 ms time window. However, frontal channels on the 800–1000 ms time window revealed a significant interaction of Cue Validity by Attractor Voice, *β* = -1.9, *SE* = .76, *t* = -2.5. To resolve the interaction, another model was constructed to test the effect of Cue Validity for the Same and for the Different Voice conditions separately. The model included random intercepts and slopes by participants and by items, and a random slope by Cue Validity. The model of the data including the Different Voice conditions revealed a significant effect of Cue Validity, *β* = -1.2, *SE* = .61, *t* = -2.0, while the model on the data including the Attractor Same Voice conditions did not reveal a significant effect of Cue Validity. The model run for posterior channels on the 800–1000 ms time window also revealed a significant interaction of Cue Validity by Attractor Voice, *β* = -2.1, *SE* = .74, *t* = -2.9. The interaction arose because the model for the Same Voice condition revealed a significant effect of Cue Validity, *β* = 1.4, *SE* = .60, *t* = 2.3, whereas the model for the Different Voice condition did not. In sum, the interaction stemmed from the greater frontal positivity for Invalid versus Valid Cue critical phrases when the Attractor Voice was Different and not when Attractor Voice was Same. This pattern of results is a form of conceptual replication of [37]. A parallel model on the 600–800 ms time window was also run. This model showed neither significant main effects of Cue Validity or Attractor Voice, nor the interaction of the two, |*t|*s < 1.

### 1.3 Discussion of Experiment 1

The pattern of results suggests that latent cue match between the cue and the most recent phrase, the attractor verb phrase, disrupts processing, but only when a grammatical antecedent is not readily elicited from memory, i.e., when the cue is invalid or ungrammatical. Such a pattern suggests that when the cues are insufficient to elicit a grammatical antecedent, that the representation with the strongest cue-match (plus recency) is disruptive to retrieval and subsequent processing, perhaps even being considered as an antecedent, although the latter is speculation. But such an interpretation of the effect would be consistent with previous literature where partial cue match in the ungrammatical situations has been argued to trigger an illusion of Cue Validity, such that the attractor representation is momentarily considered a grammatical antecedent, as in agreement attraction [30]. Another possible interpretation is that the recency of the attractor phrase and its partial cue match along a dimension that ought to be diagnostic (voice features) overrides (the actually diagnostic) structural cues at retrieval, resulting in retrieval of an ultimately illicit representation. Whether the P600 effect reflects retrieval difficulty, morphosyntactic reanalysis, or another process that is sensitive to morphosyntax, partial-cue match, and Cue Validity, is unknown.

ERPs also yield qualitative information about processing; here the distribution of the effect across the head is rather unusual when compared to classic morphosyntatic violation-related P600 effects, which tend to be more posteriorly distributed [56]. However, there is evidence in the literature for fronto-centrally distributed P600 effects associated with difficulty resolving a pronoun without an explicit antecedent [57], or resolving subject-verb relations in the face of increased discourse complexity in the form of more available referents [58]. Although also highly speculative, one possible functional interpretation of the frontal P600 effect observed here is that it indexes difficulty recovering an antecedent when the system is faced with discordant cues: a recent verb phrase shares the same voice features as the retrieval cues at the ellipsis site, but it is syntactically illicit.

In sum, partial match of a recent representation to a retrieval cue was disruptive to processing; that the effect was only observed under the presence of matching representations in the recent sentence context/ memory, in combination with an invalid retrieval cue, suggests that different information types (morphosyntactic features, structural position) can dominate or be traded off at retrieval. Integration of information types when cues are compared with the contents of memory in order to elicit the target could have produced the different patterns observed as a function of condition, which varied both the contents of memory and the status of cues at retrieval. The role of morphosyntactic features, like voice, prompts the question as to whether the trading off of cues is domain specific, and whether evidence of integration with broader cue types such as semantic content can be observed. To further test cue integration at retrieval, Experiment 2 will manipulate the plausibility of the semantic fit between the embedded subject, which is always animate, and the attractor verb phrase in the relative clause. If semantic features also affect retrieval when the cue is ultimately invalid, that would be evidence that cues of multiple, formally-distinct sources of information are integrated during retrieval.

The item set from Experiment 1 was modified to manipulate the semantic fit between the embedded subject and the attractor verb phrase. Only the conditions driving the interaction, the Attractor Different conditions, were included, such that in the manipulation of the semantic fit between an animate object and the attractor verb resulted in a 2x2 design crossing semantic fit or plausibility of the attractor phrase and Cue Validity. An effect similar to Experiment 1 showing a modulation of P600 amplitude is possible given that Cue Validity is still being manipulated, however, it is also possible that we find a modulation of the N400 component, which has been showed to be sensitive to the semantic plausibility of a gapped construction: [48] used a plausibility manipulation on gapping constructions like *Ron took/sanded the planks*, *and Bill Ø the hammer*, where more implausible interpretations (sanding the hammer) elicited larger N400 amplitudes.

## 2 Experiment 2

### 2.1 Methods

#### 2.1.1 Participants

Twenty-four British English monolingual native speakers (20 females) aged between 18 and 30 years (*M* = 21 years) participated in the experiment. All participants were right-handed and were free from neurological or language disorders. None of them have participated in the pre-test that is described in the Stimuli and Experimental Design section. Ethics approval was obtained the School of Philosophy, Psychology, and Language Sciences research ethics committee (230-1617/2). Written informed consent was obtained from participants and their data were anonymized.

#### 2.1.2 Stimuli and experimental design

The experimental stimuli consisted of 156 quadruplets like (a-d) in Table 2.

**Table 2:** 

Condition	Sentence
Valid Cue, Implausible Attractor	(a) Because Jane got the meal that was sold by the takeaway that night, John did too, as usual.
Valid Cue, Plausible Attractor	(b) Because Jane got the meal that was famous at the takeaway that night, John did too, as usual.
Invalid Cue, Implausible Attractor	(c) Because Jane got the meal that was sold by the takeaway that night, John was too, as usual.
Invalid Cue, Plausible Attractor	(d) Because Jane got the meal that was famous at the takeaway that night, John was too, as usual.

Sentences like (a-b) and (c-d) had a Valid Cue or Invalid Cue continuation respectively, which was determined by whether the auxiliary word *was* or *did* corresponded with the voice of the main verb. The verbs in the relative clause were manipulated so that the verbs could or could not combine with the second subject plausibly (*John was famous*/ *John was sold*). This manipulation was to create a Cue Validity illusion wherein readers would be more likely to incorrectly regard the ungrammatical continuation as grammatical for the plausible attractor (b & d). Half the items had a subject relative clause, and the other half an object relative clause. Thirty additional sentences were used as fillers, which consisted of 15 plausible and 15 implausible sentences.

The sentences were pre-tested to check if participants would indeed be sensitive to the manipulation of the attractor plausibility. Twenty English monolinguals were instructed to read the 186 sentences word by word presented at a rate of 500 ms, and answered a question “*What happened*?” by making a choice as quickly and accurately as possible. They were always given two choices, and the choices for the experimental sentences were between a grammatical (*John got a meal from the takeaway*.) and an ungrammatical (*John was famous / was sold at the takeaway*.) interpretation. Outliers made up 3.6% of the data, being were more than two SD away from the mean reaction time and were excluded. Participants had at least 77% of the data retained for analyses (*Mean survival rate* = 96%).

**Fig 2:**
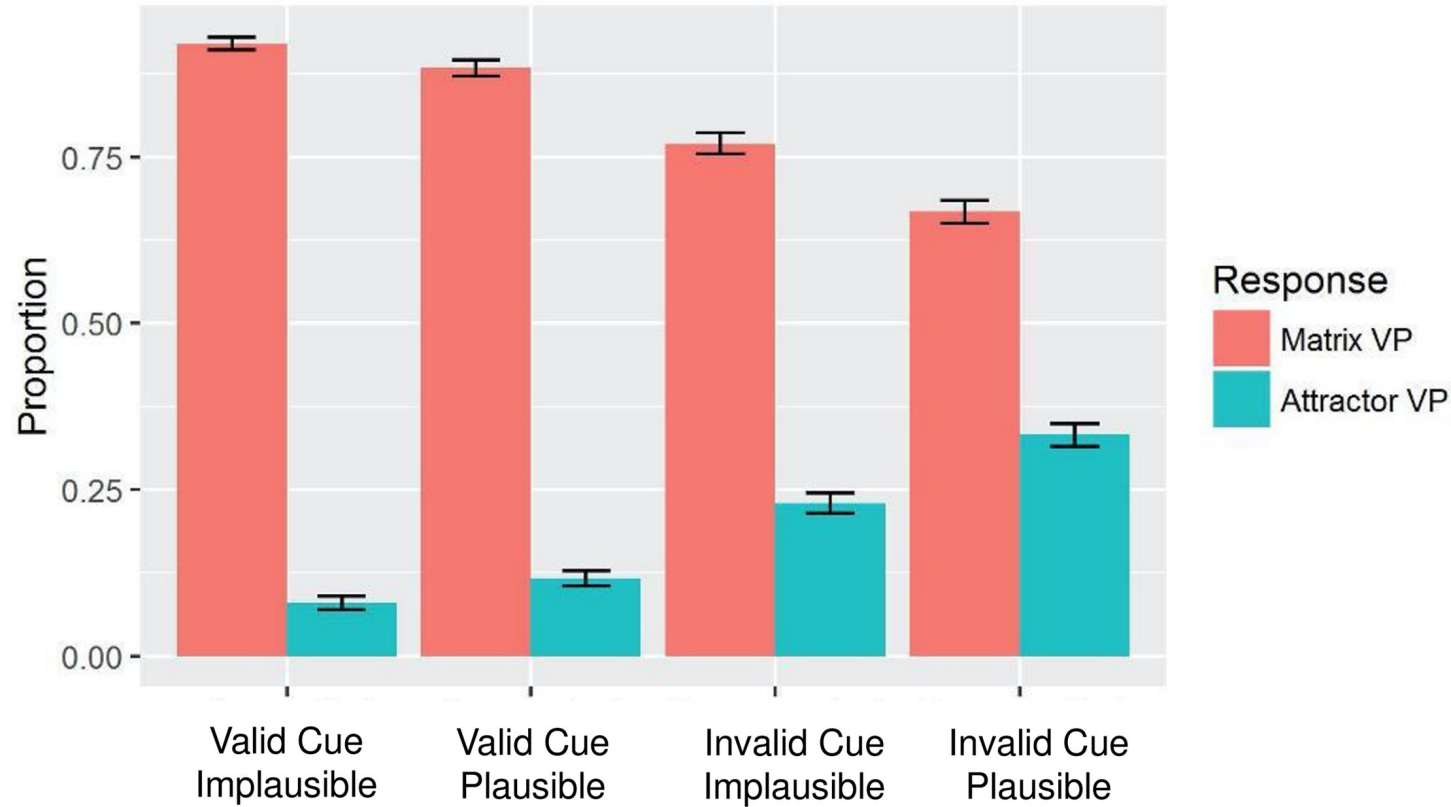
Proportion selected in 2AFC task as a function of condition.

The mean accuracy and reaction time for each condition are summarised in Table 3. I compared 2AFC judgments of the interpretations when the attractor was plausible to when the attractor was implausible (see Fig 2). The former was calculated by subtracting a standardized percentage of ungrammatical interpretation responses in (c) from the standardized percentage of grammatical interpretation responses in (a), and the latter by subtracting the standardized percentage of incorrect responses in (d) from the standardized percentage of correct responses in (b). Paired *t*-tests revealed that the discriminability between grammatical and ungrammatical interpretations of the ellipsis in the presence of an Implausible Attractor (*M* = 2.6, *SD* = 1.1) was significantly higher than that in the presence of a Plausible Attractor (*M* = 2.1, *SD* = 1.5), *t*(155) = 3.3, *p* < .001 in the item analysis, but was not significant in the participant analysis, *p* < .1. A further linear mixed effects model was constructed with the lme4 package [59] in R to evaluate the effect of Cue Validity, the effect of Attractor Plausibility and the interaction of Cue Validity by Attractor Plausibility. The model included random intercepts and slopes for Cue Validity and Attractor Plausibility by subjects and by items. The effects were regarded as significant when the associated absolute *t*-value exceeded 2 [60]. The model revealed a significant interaction of Cue Validity by Attractor Plausibility, *β* = .05, *SE* = .02, *t* = 2.2, and a significant effect of Cue Validity, *β* = -.02, *SE* = .04, *t* = -5.2. The effect of Attractor Plausibility was not significant.

**Table 3:** 

**Condition**	**a**	**b**	**c**	**d**
**Cue Validity**	Valid		Invalid	
**Attractor plausibility**	Implausible	Plausible	Implausible	Plausible
Accuracy	.92 (.27)	.88 (.32)	.77 (.42)	.67 (.47)
Discriminability	2.6 (1.1)	2.1 (1.5)		
RT	1456 (767)	1567 (809)	1778 (890)	1762 (871)

Mean accuracy and reaction time in the Cue Validity choice pre-test. *SD*s are shown in brackets.

#### 2.1.3 Procedure

The 186 items were divided into four counterbalanced lists, so that each list contained one condition per item and had the same number of sentences from each condition, and the same number of Plausible and Implausible sentences. The sentences were randomised so that no more than three sentences from the same condition appeared consecutively.

The experimental procedure was the same as in Experiment 1. The mean accuracy for comprehension questions across participants was 89% (*SD* = 8.7%, range 62.5–97.9%, 4% of the responses are excluded due to time outs). The experiment took approximately 50 minutes.

#### 2.1.4 Electroencephalogram (EEG) recording and data pre-processing

The data processing procedure was identical to that in Experiment 1, except that a standard pre-stimulus baseline (-100 to 0 ms) was used because there were no observable differences early in the epoch or in the traditional pre-stimulus baseline period. The mean proportion of artefact-free trials across conditions was 91% (*SD* = 8%), with no difference across conditions.

#### 2.1.5 Statistical analysis

An analogous statistical analysis from Experiment 1 was conducted on the time window from 300–500 ms on frontal and posterior channels in line with the prediction of an N400 effect arising from the manipulation of Attractor Plausibility. A linear mixed-effects model which evaluated the ERP amplitude predicted by the fixed effects of Cue Validity (Grammatical vs. Ungrammatical) and Attractor Plausibility (Plausible vs. Implausible), and by the interaction of the two, was built. The model additionally included random intercepts and slopes by participants and by items. Random slopes by Cue Validity and Plausibility were not included because the model with these random slopes did not converge.

### 2.2 Results

**Fig 3:**
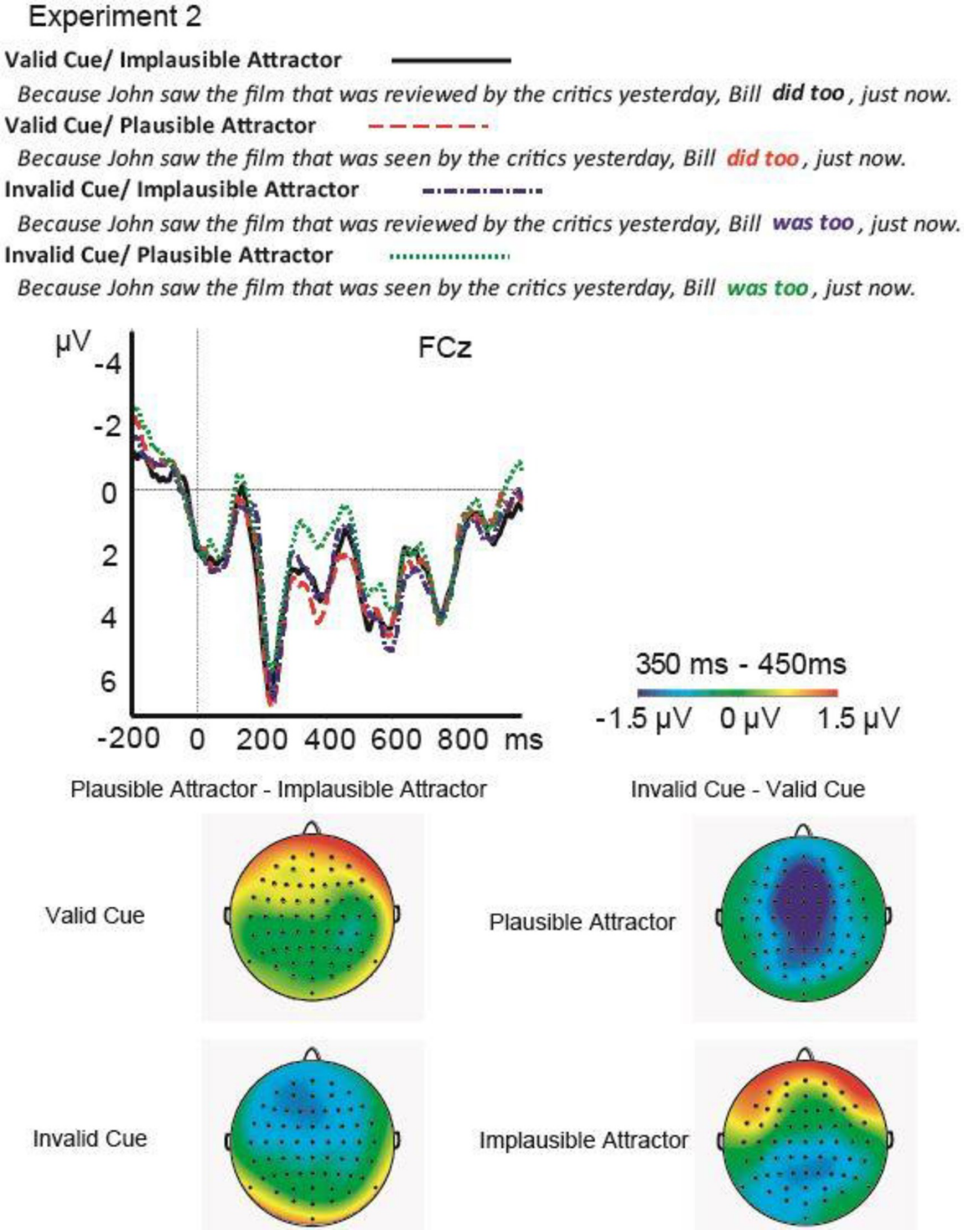
Results from Experiment 2. ERPs elicited by each condition at FCz and Pz, and the scalp distribution of the effect of Cue Validity for the Implausible and the Plausible Attractor conditions in the 300–500 ms time window befitting the N400 modulation. The pattern of ERPs on FCz is representative of the effects across frontal channels, while the pattern on Pz is representative of posterior channels.

Visual inspection of the ERP data suggests that Invalid Cue critical phrases elicited greater negativity than grammatical equivalents when the attractor was Plausible, but not when the attractor was Implausible. This negativity was most prominent at frontal channels (see Fig 3).

The model for the frontal channels on the 300–500 ms time window revealed a significant interaction of Cue Validity by Attractor Plausibility, *β* = -1.5, *SE* = .64, *t* = -2.3. The model did not show any significant main effects. To resolve the interaction, another linear mixed-effects model testing an effect of Cue Validity for Plausible and Implausible Attractor conditions was run separately. The model included random intercepts and slopes by participants and by items, and a random slope by Cue Validity. The model on the data including the Plausible Attractor conditions revealed a significant effect of Cue Validity, *β* = 1.1, *SE* = .46, *t* = 2.4, but the model including the Implausible Attractor conditions did not reveal a significant effect of Cue Validity. The model for the posterior channels on 300–500 ms time window revealed only a significant effect of Cue Validity, *β* = .7, *SE* = .32, *t* = 2.2; the interaction was not significant. To sum up, there was a greater frontal negativity for Invalid Cue versus Valid Cue critical phrases when the attractor was Plausible but not when the attractor was Implausible.

The interaction was driven by the difference between the Plausible conditions—only when the attractor could plausibly combine with the embedded subject was there a modulation of the N400. This modulation likely reflects a disruption in processing, either at retrieval of the antecedent or during integration of the embedded subject and the antecedent. As in Experiment 1, the interaction suggests that information in the recent sentence context and the information on the retrieval cues at the ellipsis site is compared or combined during sentence processing. A functional interpretation of the interaction is that the partial cue match between plausible attractors and an ungrammatical retrieval cue led to the Plausible attractor being momentarily considered as an antecedent of the ellipsis in the absence of a sufficient antecedent being elicited. Alternatively, the effect might reflect that fact that the system is sensitive to semantic similarity, even across syntactically illicit relations, and this sensitivity is only measurable when no grammatical antecedent is elicited.

## General discussion

How are linguistic representations organized in memory during ongoing sentence processing, and what information is relevant during the retrieval and interpretation of “missing” representations? The pattern of results from Experiments 1 and 2 showed evidence suggesting that information available at retrieval, that is, both information in memory *and* retrieval cues, interacts. The interaction shaped processing outcomes such that the pattern of results across both experiments is consistent with an architecture where the contents of memory are linearly combined with cues at retrieval to elicit the antecedent, providing a representation for interpretation in a new sentence position and referential relation. Information carried by a syntactically illicit ‘attractor’ representation disrupted retrieval of the antecedent and/or interpretation of the ellipsis, but only in the presence of a retrieval cue that was insufficient or ungrammatical. Disruption occurred from both of the levels of linguistic representation that were tested: In Experiment 1, when morphosyntactic voice features on the ‘attractor’ verb matched an insufficient ungrammatical retrieval cue, they disrupted processing compared to when those features did not match the cue. In Experiment 2, the plausibility of the semantic fit between the ‘attractor’ verb in the relative clause and an animate object disrupted processing when that fit was plausible compared to when it was implausible. Both of these findings suggest that information at retrieval, in the form of integrated cues, must have interacted with the contents of memory in order to produce the observed pattern of results.

Furthermore, across the two experiments, the manipulation of linguistic features of the attractor controlled the expression of the interference effect in terms of ERP componentry: manipulation of morphosyntax led to a modulation of the P600 component, while manipulation of plausibility led to an N400 effect, but the nature of the modulation was similar—an interaction drive by conditions of comparable cue status (both the Different Voice Attractor and the Plausible Attractor partially matched their respective Invalid Cues) across levels of linguistic analysis. This fact in and of itself is novel in the literature–I am aware of no other study that reports a form of replicated effect expressed on two different ERP components [20] where interference effects were observed both a sustained negativity and a P600 effect were in the same experiment, although among different groups of participants]. The paucity of cases in the literature where two different components show similar (interference) effects may also stem from the fact that there are not many ERP studies that use a similar paradigm, nor many on ellipsis. I am not aware of another ERP study which manipulates morphosyntax or plausibility of syntactically illicit attractor representations during dependency resolution other than [19, 20]. The expression of the effects may also have interesting implications for the functional interpretation of ERP components, but the current functional interpretations of the N400 and P600 components are predicated on experimental circumstances that are so distant from this ellipsis paradigm that I hesitate to speculate about componentry differences further. Nonetheless, that interaction-based interference effects can be elicited on different ERP components as a function of the type of information manipulated does suggest that ERP interference paradigms [19, 20] tap into processing at the same granularity or ‘carving joints’ as ERP components do. The difference in elicited componentry can arguably be said to be a function of the level of linguistic analysis over which cues and the contents of memory interact.

The distribution of the effects was frontal in both experiments, regardless of the different components that were elicited, and in contrast with most language-elicited P600 and N400 effects. In sentence processing experiments, frontal P600 effects have been associated with resolving who did what to whom in the face of multiple referents in a discourse [58] and with processing morphosyntactic agreement violations that have consequences for the possible reanalysis or repair of "who did what to whom" following the violation [61]. Both of these functional speculations relate to processing circumstances present in Experiment 1, namely, that given an ungrammatical ellipsis/ insufficient retrieval cue to the antecedent, representations in memory that also provide a partial match to the retrieval cues (i.e., the attractor that matched in voice features to the retrieval cue) might also be called upon until the insufficiency of those representations becomes apparent and other interpretations are computed or considered. In addition to featuring morphosyntactic violations, the Ungrammatical conditions in Experiment 1 also featured a form of referential insufficiency or failure, whereby the cues to the antecedent were not diagnostic. This situation might be similar to the processing circumstances in [58] where multiple referents were available, but further computation was needed determine who did what to whom in the face of ambiguity.

In recognition memory, frontal N400s have been elicited in paradigms that vary the familiarity of a stimulus [62], but these effects have been argued to reflect a form of conceptual priming that varies during the processing of old versus new stimuli, rather than familiarity per se [63]. In some sense, ellipsis calls on a previously processed "old" representation in a "new" way, but given that it is not clear that frontal N400s are functionally or even descriptively separate from more posteriorly distributed N400s [45]. The anteriority of the results in Experiment 2 may result from the partial match between an insufficient or ungrammatical cue and an attractor. The partial match might lead to sub-threshold activation of a representation in a similar way that judgments of familiarity can be said to be a lesser form of representational activation compared to full recognition or recollection.

### Cue integration via a linear weighting scheme—a function of how relevant information is in encoded in a language?

In both experiments, it is possible to interpret the results as a sort of ‘reanalysis’ effect, where the ‘correct’ representation comes to mind despite (or because of) the invalid or ungrammatical cue. For example, that the observed interactions were driven by conditions with cues that were insufficient to elicit a grammatical antecedent for the ellipsis could be mean that the observed results reflect processing in the system when things ‘go wrong’, rather than what the system ‘normally does.’ While this is certainly a limitation for interpretation of data stemming from all violation paradigms, such a paradigm was needed in order to allow the manipulation of information both before and at the onset of retrieval. Manipulating information both in memory (the attractor) and at retrieval (the Cue Validity/validity of the cue) can reveal how the system weights retrieval cues to information in memory. Despite these caveats, the findings reported here can be interpreted such that they make two substantive claims about how cues are integrated during language comprehension.

Most models of recognition memory operate on global matching of cues to memory at retrieval such that all available cues are utilized [64]. Since no special status is given as a function of location or temporal distribution, retrieval cues are likely to be combined dynamically as information processing goes forward, though the computational mechanisms that govern this process for language processing are only beginning to be investigated [5, 11,13]. In recognition memory and perception, most models posit cue combination via a linear weighting scheme [65]. A linear cue-weighting combination scheme would mean that different sources of information can have a range of values, and those with extreme values could either dominate processing or be completely discounted. As such, a caveat in discriminating between linear and non-linear systems comes from the fact that linear cue-weighting can mimic non-linear schemes. Non-linear schemes restrict the retrieval architecture by forcing a processing “bottle neck,” formally-equivalent to a gating function, whereby perceptual features in certain configurations or distributions do not produce interference because they are not considered or sampled from [65]. In language processing context, non-linear cue weighting would imply that representations in certain structural positions or of certain syntactic categories do not produce interference because they are not considered, or, in other words, that *only* representations in licensed syntactic positions create interference [13]. Thus, falsifying a non-linear scheme would entail observing interference from syntactically illicit representations, as reported here and as a few other recent behavioural and ERP studies have shown [13, 14, 19, 20, 22, 30, 32]. That is, the data reported here arguably rule out (barring the mimicry caveat) a non-linear weighting scheme because they show interference from representations irrespective of structural position—although it is still possible, and I believe quite likely even, that attractors in licit structural positions should be *more disruptive* than attractors in illicit ones—this question remains to be investigated. In contrast to previous work from our lab showing that grammatical noun-phrase ellipsis in Spanish was vulnerable to interference from representations in syntactically illicit positions, only ungrammatical cues/ellipses were vulnerable to interference in the experiments reported in this paper [19, 20]. In [30], the authors make the useful distinction between effects on ungrammatical sentences, which are classified as agreement attraction effects, and effects on grammatical sentences which are classified as interference effects. I concede the effects reported here thus can be seen as agreement attraction effects, but I insist on parsimony of mechanism and computational principles that give rise to them: cue integration and interference. One clear hypothesis as to why these differences were observed is that cue combination weights differ by language–just as languages naturally distribute cue information differently across grammatical systems [66], so vary the cue weighting schemes [5, 13]. In Spanish, grammatical gender agreement is a robust cue for anaphoric and other dependencies, and recent evidence suggests that agreement information is projected forward during phrasal processing [43]. The distribution of information and mapping between these distributions and cue types in Spanish and English are likely to differ substantially, but thus far, evidence suggests that the weighting scheme is likely to be linear. The generation of interference effects from syntactically illicit positions does not necessarily imply or even suggest that syntactic structure does not play a crucial role in ongoing language processing. Rather, it indicates that the nature of the (measurable) online recruitment and integration of linguistic cues is still beyond our understanding.

### A linear cue-weighting scheme and the nature of the retrieval operation

A second theoretical interpretation of the findings reported here regards the computational mechanism by which cues directly elicit memory representations during retrieval. Models of recognition memory have long posited convolution as the formal operation by which cues and the contents of memory are combined to form a new memory trace [67–70]. However, convolution has a major computational drawback: it loses the discrete representation of its input polynomials, which could only be recovered via a de-convolution operation. Any product-based operation or representation, including tensor products, a current popular solution in connectionist modelling, will have similar information loss problems (see [71] for discussion). While information loss from multiplicative operations might suffice, or even be desirable, for perception and memory architectures, such a principle is, at least on some level, patently insufficient for human language. At bare minimum, language processing requires simultaneous representation of discrete input units and compositional, hierarchical output representations at multiple timescales. Another candidate mechanism for the direct-access retrieval operation that is not vulnerable to such information loss is the super-position of cues on memory, a form of vector addition. In a vector system, representational independence can be preserved while coding various sources of cue information in a recoverable way if the mechanism is additive rather than multiplicative, making vector addition an attractive candidate for the implementation of direct-access retrieval [71, 72]. To the extent that the data reported here support the cue integration hypothesis, they also, by inductive inference, implicate a computational mechanism for direct-access retrieval that has properties of vector addition.

Given that it is likely that multiple types of linguistic representations could serve as retrieval cues, *how* these representations might be combined or integrated in order to probe memory becomes mechanistically relevant. In perception, psychophysics and basic memory research, multimodal cues are combined and integrated as a standard architectural principle [65, 70, 73]. Within a broader perceptual processing framework where cue integration is the primary mechanistic operation, vectors representing the feature values of a given cue are summated with each other and normalized by that cue’s reliability, an estimate of uncertainty about the reliability of a cue to a given property of the environment [5, 73]. The pattern of results from the experiments presented here is consistent with such a mechanism subserving sentence comprehension.

## Supporting information

S1 Table Experimental Stimuli for Experiments 1 and 2. (DOCX)

S1 Fig All electrodes Experiment 1. (TIF)

S2 Fig All electrodes Experiment 2. (TIF)
