# Supplementary material for: Cue integration during sentence comprehension: Electrophysiological evidence from ellipsis
Source: PLoS One. 2018 Nov 29;13(11):e0206616. doi: 10.1371/journal.pone.0206616 (PMC6264514; doi:10.1371/journal.pone.0206616)

Supporting Information

Critical sentences for Experiment 1.

| Item | Voice | Sentence |
| --- | --- | --- |
| 1 | same | Because John saw the film that the critics reviewed yesterday, Bill did too/was too, just now. |
|  | different | Because John saw the film that was reviewed by the critics yesterday, Bill did too/was too, just now. |
| 2 | same | Because Jim was excited about the book that was praised by critics in the ad, Rob was too/did too, after all. |
|  | different | Because Jim was excited about the book that critics praised in the ad, Rob was too/did too, after all. |
| 3 | same | Because Tom liked the cake that the chef prepared earlier, Jack did too/was too, quite expectedly. |
|  | different | Because Tom liked the cake that was prepared by the chef earlier, Jack did too/was too, quite expectedly. |
| 4 | same | Because Will was tempted by the cake that the café displayed last week, Carl was too/did too, after lunch. |
|  | different | Because Will was tempted by the cake that was displayed by the café last week, Carl was too/did too, after lunch. |
| 5 | same | Since Sally bought the perfume that the actress endorsed on TV, Holly did too/was too, on sale. |
|  | different | Since Sally bought the perfume that was endorsed by the actress on TV, Holly did too/was too, on sale. |
| 6 | same | Since Sue was happy with the dresses that were altered by the tailor yesterday, Eva was too/did too, as expected. |
|  | different | Since Sue was happy with the dresses that the tailor altered yesterday, Eva was too/did too, as expected. |
| 7 | same | After Lee completed the essay that the lecturer assigned last week, Seamus did too/was too, at home. |
|  | different | After Lee completed the essay that was assigned by the lecturer last week, Seamus did too/was too, at home. |
| 8 | same | After Liam was praised for work that was marked by the invigilator yesterday, Lewis was too/did too, on Friday. |
|  | different | After Liam was praised for work that the invigilator marked yesterday, Lewis was too/did too, on Friday. |
| 9 | same | Whenever Toni read novels that the book club endorsed recently, Anne did too/was too, without complaining. |
|  | different | Whenever Toni read novels that were endorsed by the book club recently, Anne did too/was too, without complaining. |
| 10 | same | Whenever Tamsin was stressed about the party that was planned by the employees at work, Hannah was too/did too, despite tranquilizers. |
|  | different | Whenever Tamsin was stressed about the party that the employees planned at work, Hannah was too/did too, despite tranquilizers. |
| 11 | same | Since Sandy bought the tickets that the theatre advertised in the magazine, Bill did too/was too, rather reluctantly. |
|  | different | Since Sandy bought the tickets that were advertised by the theatre in the magazine, Bill did too/was too, rather reluctantly. |
| 12 | same | Since Chloe was scared by the knife that the butcher held that night, Sam was too/did too, quite rightly. |
|  | different | Since Chloe was scared by the knife that was held by the butcher that night, Sam was too/did too, quite rightly. |
| 13 | same | Because Georgia accepted the flowers that the benefactor sent in the post, Hannah did too/was too, somewhat reluctantly. |
|  | different | Because Georgia accepted the flowers that were sent by the benefactor in the post, Hannah did too/was too, somewhat reluctantly. |
| 14 | same | Because Gina was unimpressed by the wine that was served by the waiter at lunch, Erik was too/did too, as always. |
|  | different | Because Gina was unimpressed by the wine that the waiter had served at lunch, Erik was too/did too, as always. |
| 15 | same | Because Philip rejected the offer that the negotiator proposed in the meeting, Danny did too/was too, that afternoon. |
|  | different | Because Philip rejected the offer that was proposed by the negotiator in the meeting, Danny did too/was too, that afternoon. |
| 16 | same | Because Arthur was ridiculed by the article that the blogger wrote online, Anna was too/did too, sadly enough. |
|  | different | Because Arthur was ridiculed by the article that was written by the blogger online, Anna was too/did too, sadly enough. |
| 17 | same | As Jessica evaluated the transcripts that the student submitted to the journal, Rachel did too/was too, to double-check. |
|  | different | As Jessica evaluated the transcripts that were submitted by the student to the journal, Rachel did too/was too, to double-check. |
| 18 | same | Because Jane was interested in the car that was advertised by the woman online, Ellen was too/did too, causing problems. |
|  | different | Because Jane was interested in the car that the woman advertised online, Ellen was too/did too, causing problems. |
| 19 | same | Because Pete liked the band that the radio presenter interviewed this morning, Todd did too/was too, quite surprisingly. |
|  | different | Because Pete liked the band that were interviewed by the radio presenter this morning, Todd did too/was too, quite surprisingly. |
| 20 | same | Because Simon was shocked by the painting that the artist unveiled earlier, Richard was too/did too, rather clearly. |
|  | different | Because Simon was shocked by the painting that was unveiled by the artist earlier, Richard was too/did too, rather clearly. |
| 21 | same | Since Leah completed the crossword that the newspaper published yesterday, Cassie did too/was too, during lunch. |
|  | different | Since Leah completed the crossword that was published by the newspaper yesterday, Cassie did too/was too, during lunch. |
| 22 | same | Since Amy was upset about the trap that the farmer set last night, Kelly was too/did too, quite understandably. |
|  | different | Since Amy was upset about the trap that was set by the farmer last night, Kelly was too/did too, quite understandably. |
| 23 | same | As Cleo ran the race that the trainer organised last summer, Emma did too/was too, somewhat reluctantly. |
|  | different | As Cleo ran the race that was organised by the trainer last summer, Emma did too/was too, somewhat reluctantly. |
| 24 | same | As Lena was seen by the camera that policemen fitted in the cabinet, Laura was too/did too, moments later. |
|  | different | As Lena was seen by the camera that was fitted by policemen in the cabinet, Laura was too/did too, moments later. |
| 25 | same | As Beth phoned the decorator that the neighbours recommended over lunch, Anna did too/was too, right away. |
|  | different | As Beth phoned the decorator that was recommended by the neighbours over lunch, Anna did too/was too, right away. |
| 26 | same | As Camille was intrigued by the rattling that was produced by the parcel outside, Mia was too/did too, at once. |
|  | different | As Camille was intrigued by the rattling that the parcel produced outside, Mia was too/did too, at once. |
| 27 | same | Because Chelsea wrapped the presents that the children bought on Saturday, Jo did too/was too, in paper. |
|  | different | Because Chelsea wrapped the presents that were bought by the children on Saturday, Jo did too/was too, in paper. |
| 28 | same | Because Gemma was invited to the hotel that the journalist filmed on TV, Holly was too/did too, months later. |
|  | different | Because Gemma was invited to the hotel that was filmed by the journalist on TV, Holly was too/did too, months later. |
| 29 | same | Since Alexa stole the sweets that the shopkeeper displayed on the shelf, Lisbeth did too/was too, after hesitating. |
|  | different | Since Alexa stole the sweets that were displayed by the shopkeeper on the shelf, Lisbeth did too/was too, after hesitating. |
| 30 | same | Since Marjory was annoyed by the ball that was kicked by youths in the street, Catherine was too/did too, but less. |
|  | different | Since Marjory was annoyed by the ball that youths kicked in the street, Catherine was too/did too, but less. |
| 31 | same | Because Joe entered the competition that the teacher created yesterday, Richard did too/was too, but reluctantly. |
|  | different | Because Joe entered the competition that was created by the teacher yesterday, Richard did too/was too, but reluctantly. |
| 32 | same | Because Jamie was disliked by the team that was sponsored by businessmen in the town, Felix was too/did too, even more. |
|  | different | Because Jamie was disliked by the team that businessmen sponsored in the town, Felix was too/did too, even more. |
| 33 | same | Since Jen ate the curry that the vendor prepared on the hob, Hannah did too/was too, as dinner. |
|  | different | Since Jen ate the curry that was prepared by the vendor on the hob, Hannah did too/was too, as dinner. |
| 34 | same | Since Sally was frightened by the costume that the clown wore that evening, Nina was too/did too, quite understandably. |
|  | different | Since Sally was frightened by the costume that was worn by the clown that evening, Nina was too/did too, quite understandably. |
| 35 | same | As Mike attended the meeting that the company scheduled last week, Ryan did too/was too, as always. |
|  | different | As Mike attended the meeting that was scheduled by the company last week, Ryan did too/was too, as always. |
| 36 | same | When Bill was disgusted by the tooth that the dentist removed recently, John was too/did too, at once. |
|  | different | When Bill was disgusted by the tooth that was removed by the dentist recently, John was too/did too, at once. |
| 37 | same | Because Harriet wore her uniform that the cleaner ironed yesterday, Felicity did too/was too, after deliberation. |
|  | different | Because Harriet wore her uniform that was ironed by the cleaner yesterday, Felicity did too/was too, after deliberation. |
| 38 | same | Because Helen was alarmed by a siren that the ambulance sounded outside, Felicity was too/did too, while reading. |
|  | different | Because Helen was alarmed by a siren that was sounded by the ambulance outside, Felicity was too/did too, while reading. |
| 39 | same | While Jeremy drew the sunset that the photograph captured last night, Declan did too/was too, quite beautifully. |
|  | different | While Jeremy drew the sunset that was captured by the photograph last night, Declan did too/was too, quite beautifully. |
| 40 | same | While Richard was licked by the dog that the vet checked today, Declan was too/did too, at home. |
|  | different | While Richard was licked by the dog that was checked by the vet today, Declan was too/did too, at home. |
| 41 | same | Since Keira followed the car that the mayor drove on Saturday, Hannah did too/was too, at night. |
|  | different | Since Keira followed the car that was driven by the mayor on Saturday, Hannah did too/was too, at night. |
| 42 | same | Since Elizabeth was enticed by the dress that the magazine styled this month, Hannah was too/did too, quite understandably. |
|  | different | Since Elizabeth was enticed by the dress that was styled by the magazine this month, Hannah was too/did too, quite understandably. |
| 43 | same | Because Paul washed the bike that his parents bought this Christmas, Neil did too/was too, just outside. |
|  | different | Because Paul washed the bike that was bought by his parents this Christmas, Neil did too/was too, just outside. |
| 44 | same | Because Jake was woken by yelling that the children did in the street, Neil was too/did too, that morning. |
|  | different | Because Jake was woken by yelling that was done by the children in the street, Neil was too/did too, that morning. |
| 45 | same | Because Fred denounced the article that the reviewers rejected yesterday, Murray did too/was too, at last. |
|  | different | Because Fred denounced the article that was rejected by the reviewers yesterday, Murray did too/was too, at last. |
| 46 | same | Because George was angered by the article that was written by the journalist online, Abe was too/did too, very predictably. |
|  | different | Because George was angered by the article that the journalist wrote online, Abe was too/did too, very predictably. |
| 47 | same | Because Chloe distrusted the brand that the shop sold enthusiastically, Sarah did too/was too, without question. |
|  | different | Because Chloe distrusted the brand that was sold by the shop enthusiastically, Sarah did too/was too, without question. |
| 48 | same | Because Diana was interested in the concept that was explained by the scientist after all, Jessica was too/did too, later on. |
|  | different | Because Diana was interested in the concept that the scientist explained after all, Jessica was too/did too, later on. |
| 49 | same | Since Katherine went to the art fair that the school sponsored frequently, Lynn did too/was too, on Monday. |
|  | different | Since Katherine went to the art fair that was sponsored at school frequently, Lynn did too/was too, on Monday. |
| 50 | same | Since Emily was interested in visiting the exhibition that was mentioned at artist frequently, Brenda was too/did too, pretty much. |
|  | different | Since Emily was interested in visiting the exhibition that the artist mentioned frequently, Brenda was too/did too, pretty much. |
| 51 | same | As Dylan played the game that his friend had mentioned at lunchtime, Will did too/was too, on Thursday. |
|  | different | As Dylan played the game that was mentioned by his friend at lunchtime, Will did too/was too, on Thursday. |
| 52 | same | As Avery was delighted by the concert that was organised by girls on Friday, Jake was too/did too, pretty much. |
|  | different | As Avery was delighted by the concert that girls had organised on Friday, Jake was too/did too, pretty much. |
| 53 | same | Because Kim accepted the wine that her mum gave them yesterday, Chris did too/was too, as usual. |
|  | different | Because Kim accepted the wine that was given by her mum yesterday, Chris did too/was too, as usual. |
| 54 | same | Because Nina was unhappy about the gift that was given by her mum earlier, Mike was too/did too, as expected. |
|  | different | Because Nina was unhappy about the gift that her mum gave them earlier, Mike was too/did too, as expected. |
| 55 | same | While Tim read the book that the class required for the semester, Alice did too/was too, yesterday evening. |
|  | different | While Tim read the book that was required by the class for the semester, Alice did too/was too, yesterday evening. |
| 56 | same | While Alexander was annoyed by the project that was required by examiners as coursework, Matilde was too/did too, even more. |
|  | different | While Alexander was annoyed by the project that examiners required as coursework, Matilde was too/did too, even more. |
| 57 | same | Since Harry stole the whiskey that his dad had opened recently, Rachel did too/was too, on Saturday. |
|  | different | Since Harry stole the whiskey that was opened by his dad recently, Rachel did too/was too, on Saturday. |
| 58 | same | Since Colm was envious of the car that was bought by his stepdad recently, Isabelle was too/did too, pretty much. |
|  | different | Since Colm was envious of the car that his stepdad bought recently, Isabelle was too/did too, pretty much. |
| 59 | same | Because Nikki listened to the DJ that her brother suggested on the motorway, Rosie did too/was too, after all. |
|  | different | Because Nikki listened to the DJ that was suggested by her brother on the motorway, Rosie did too/was too, after all. |
| 60 | same | Because Terry was unsure about the music that was suggested by bloggers yesterday, Pamela was too/did too, as always. |
|  | different | Because Terry was unsure about the music that bloggers suggested yesterday, Pamela was too/did too, as always. |
| 61 | same | As Maria chose the most expensive wine that the restaurant served that night, Ben did too/was too, as always |
|  | different | As Maria chose the most expensive wine that was served by the restaurant that night, Ben did too/was too, as always |
| 62 | same | As Judith was impressed by the cheese that was imported by the restaurant recently, Johnny was too/did too, rather unexpectedly. |
|  | different | As Judith was impressed by the cheese that the restaurant imported recently, Johnny was too/did too, rather unexpectedly. |
| 63 | same | While Claire mentioned vaccinations that the family needed on holiday, Carolyn did too/was too, next door. |
|  | different | While Claire mentioned vaccinations that were needed by the family on holiday, Carolyn did too/was too, next door. |
| 64 | same | While Tessa was packing clothes that were needed by her daughter on holiday, Ruby was too/did too, next door. |
|  | different | While Tessa was packing clothes that her daughter needed on holiday, Ruby was too/did too, next door. |
| 65 | same | Since Steven ate the cake that his daughter had made for his birthday party, Taylor did too/was too, when offered. |
|  | different | Since Steven ate the cake that was made by his daughter for his birthday party, Taylor did too/was too, when offered. |
| 66 | same | Since Rene was obliged to eat the meal that was made by his son recently, Dan was too/did too, when offered. |
|  | different | Since Rene was obliged to eat the meal that his son had made recently, Dan was too/did too, when offered. |
| 67 | same | Because Martha decided to visit the cafe that the magazine advertised often, Becky did too/was too, that day. |
|  | different | Because Martha decided to visit the cafe that was advertised by the magazine often, Becky did too/was too, that day. |
| 68 | same | Because Vicki was aware of the deal that was advertised by the magazine often, Norma was too/did too, as usual. |
|  | different | Because Vicki was aware of the deal that the magazine advertised often, Norma was too/did too, as usual. |
| 69 | same | As Joe gave money to the charity that the school supported occasionally, Sally did too/was too, to help. |
|  | different | As Joe gave money to the charity that was supported by the school occasionally, Sally did too/was too, to help. |
| 70 | same | As Armin was drafted by companies that were associated with alumni regularly, Lorelai was too/did too, surprisingly enough. |
|  | different | As Armin was drafted by companies that alumni associated with regularly, Lorelai was too/did too, surprisingly enough. |
| 71 | same | Since Tina liked the coach that the team hired in the new year, Clara did too/was too, after all. |
|  | different | Since Tina liked the coach that was hired by the team in the new year, Clara did too/was too, after all. |
| 72 | same | Since Nicole was liked by the coach that was hired by the team last month, Mary was too/did too, after all. |
|  | different | Since Nicole was liked by the coach that the team hired last month, Mary was too/did too, after all. |
| 73 | same | While Andy got ready for the party that Facebook planned recently, Edward did too/was too, at home. |
|  | different | While Andy got ready for the party that was planned on Facebook recently, Edward did too/was too, at home. |
| 74 | same | While Michael was preparing the conference that was planned by managers annually, Luke was too/did too, to help. |
|  | different | While Michael was preparing the conference that managers planned annually, Luke was too/did too, to help. |
| 75 | same | When Kelly entered the marathon that her town held again, Ollie did too/was too, at last. |
|  | different | When Kelly entered the marathon that was held by her town again, Ollie did too/was too, at last. |
| 76 | same | When Cathy was excited by the rally that was held by the town again, Brian was too/did too, rather unsurprisingly. |
|  | different | When Cathy was excited by the rally that the town held again, Brian was too/did too, rather unsurprisingly. |
| 77 | same | As Emma went to the exercise class that the doctor suggested yesterday, Carina did too/was too, without registering. |
|  | different | As Emma went to the exercise class that was suggested by the doctor yesterday, Carina did too/was too, without registering. |
| 78 | same | As Rose was excited to try the physiotherapy that was suggested by the doctor yesterday, Gloria was too/did too, but briefly. |
|  | different | As Rose was excited to try the physiotherapy that the doctor suggested yesterday, Gloria was too/did too, but briefly. |
| 79 | same | Because Matt got a meal that the take-away promoted that night, Andrea did too/was too, not long after. |
|  | different | Because Matt got a meal that was promoted by the take-away that night, Andrea did too/was too, not long after. |
| 80 | same | Because Robert was reassured by the babysitter that was with the children that night, Kelly was too/did too, of course. |
|  | different | Because Robert was reassured by the babysitter that the children were with that night, Kelly was too/did too, of course. |
| 81 | same | Because Peter went to the only comedy that the cinema showed that week, Simon did too/was too, that night. |
|  | different | Because Peter went to the only comedy that was shown by the cinema that week, Simon did too/was too, that night. |
| 82 | same | Because Ian was impressed by the comedy that was shown by cinemas lately, Noah was too/did too, as always. |
|  | different | Because Ian was impressed by the comedy that cinemas showed lately, Noah was too/did too, as always. |
| 83 | same | As Daisy criticised the game that her son received as present yesterday, Hannah did too/was too, right away. |
|  | different | As Daisy criticised the game that was received by her son as present yesterday, Hannah did too/was too, right away. |
| 84 | same | As Caroline was dismayed by the toy gun that was bought by her son yesterday, Tracy was too/did too, right away. |
|  | different | As Caroline was dismayed by the toy gun that her son bought yesterday, Tracy was too/did too, right away. |
| 85 | same | While Max listened to the album that the band released recently, Tim did too/was too, while driving. |
|  | different | While Max listened to the album that was released by the band recently, Tim did too/was too, while driving. |
| 86 | same | While Barry was in line for the gadget that was on offer at Tesco recently, Maxim was too/did too, quite unusually. |
|  | different | While Barry was in line for the gadget that Tesco had on offer recently, Maxim was too/did too, quite unusually. |
| 87 | same | Since Siobhan welcomed the colleague that the office hired last week, Duncan did too/was too, but reluctantly. |
|  | different | Since Siobhan welcomed the colleague that was hired by the office last week, Duncan did too/was too, but reluctantly. |
| 88 | same | Since Cyrus was nasty to the colleague that was hired by the office last week, Bradley was too/did too, quite reflexively. |
|  | different | Since Cyrus was nasty to the colleague that the office hired last week, Bradley was too/did too, quite reflexively. |
| 89 | same | When Rebecca went to the house that her grandma rented during summer, Claudia did too/was too, to relax. |
|  | different | When Rebecca went to the house that was rented by her grandma during summer, Claudia did too/was too, to relax. |
| 90 | same | When Penny was worried about her dog who was attacked often, Melinda was too/did too, of course. |
|  | different | When Penny was worried about her dog who attacked often, Melinda was too/did too, of course. |
| 91 | same | Since Anna chose the cake that the chef offered for dessert, Kerry did too/was too, while smiling. |
|  | different | Since Anna chose the cake that was offered by the chef for dessert, Kerry did too/was too, while smiling. |
| 92 | same | Since Salome was intrigued by the story that was told by the refugee in the report, Mark was too/did too, but hesitated. |
|  | different | Since Salome was intrigued by the story that the refugee told in the report, Mark was too/did too, but hesitated. |
| 93 | same | As Rob took part in the protest that the society ran for charity, Freya did too/was too, after all. |
|  | different | As Rob took part in the protest that was run by the society for charity, Freya did too/was too, after all. |
| 94 | same | As Ronnie was indignant about the scheme that was run by charities recently, Heather was too/did too, quite fairly. |
|  | different | As Ronnie was indignant about the scheme that charities ran recently, Heather was too/did too, quite fairly. |
| 95 | same | Because Tyler climbed the glacier that experts praised this year, Gregg did too/was too, with her. |
|  | different | Because Tyler climbed the glacier that was praised by experts this year, Gregg did too/was too, with her. |
| 96 | same | Because Kenneth was in awe of the mountain that was classified by experts as challenging, Patrick was too/did too, not surprisingly. |
|  | different | Because Kenneth was in awe of the mountain that experts classified as challenging, Patrick was too/did too, not surprisingly. |
| 97 | same | While Hamish cleaned the flat for the inspection that landlords did monthly, Ally did too/was too, very quickly. |
|  | different | While Hamish cleaned the flat for the inspection that was done by landlords monthly, Ally did too/was too, very quickly. |
| 98 | same | Since Kevin was prepared for the inspection that was run by managers monthly, Jordan was too/did too, with reluctance. |
|  | different | Since Kevin was prepared for the inspection that managers ran monthly, Jordan was too/did too, with reluctance. |
| 99 | same | As Terry made biscuits that the neighbours coveted again, Eva did too/was too, to help. |
|  | different | As Terry made biscuits that were coveted by the neighbours again, Eva did too/was too, to help. |
| 100 | same | As Susie was enlisted for the event that was organised by villagers again, Roxanne was too/did too, to help. |
|  | different | As Susie was enlisted for the event that villagers organised again, Roxanne was too/did too, to help. |
| 101 | same | When Kyle considered the computer that the report recommended lately, Nick did too/was too, but less. |
|  | different | When Kyle considered the computer that was recommended by the report lately, Nick did too/was too, but less. |
| 102 | same | When Jerry was spammed by the website that was mentioned by the hacker repeatedly, Ethan was too/did too, at once. |
|  | different | When Jerry was spammed by the website that the hacker mentioned repeatedly, Ethan was too/did too, at once. |
| 103 | same | Because Harriet went to the protest that the students planned yesterday, Holly did too/was too, for support. |
|  | different | Because Harriet went to the protest that was planned by the students yesterday, Holly did too/was too, for support. |
| 104 | same | Because Melanie was excited about the rally that was organised by protestors this weekend, Jean was too/did too, for sure. |
|  | different | Because Melanie was excited about the rally that protestors organised this weekend, Jean was too/did too, for sure. |
| 105 | same | While Amy washed the clothes that the models wore on the catwalk yesterday, Vicky did too/was too, very carefully. |
|  | different | While Amy washed the clothes that were worn by the models on the catwalk yesterday, Vicky did too/was too, very carefully. |
| 106 | same | While Cecily was stressed by the essay that the teacher set last week, Scarlett was too/did too, at home. |
|  | different | While Cecily was stressed by the essay that was set by the teacher last week, Scarlett was too/did too, at home. |
| 107 | same | Because Becky drank the tea that the waitress made minutes earlier, Erin did too/was too, right away. |
|  | different | Because Becky drank the tea that was made by the waitress minutes earlier, Erin did too/was too, right away. |
| 108 | same | Because Shauna was mocked by the gang that the shopkeeper reported last week, Nancy was too/did too, yet again. |
|  | different | Because Shauna was mocked by the gang that was reported by the shopkeeper last week, Nancy was too/did too, yet again. |
| 109 | same | Since John ate the curry that the waiter recommended yesterday, Sophie did too/was too, but reluctantly. |
|  | different | Since John ate the curry that was recommended by the waiter yesterday, Sophie did too/was too, but reluctantly. |
| 110 | same | Since Paul was delayed by the flight that the pilot navigated in blizzard, Christine was too/did too, once again. |
|  | different | Since Paul was delayed by the flight that was navigated by the pilot in blizzard, Christine was too/did too, once again. |
| 111 | same | When Beth wanted the job that the company offered in accounting, Anna did too/was too, completely unexpectedly. |
|  | different | When Beth wanted the job that was offered by the company in accounting, Anna did too/was too, completely unexpectedly. |
| 112 | same | When Deborah was excited by the flat that the agency advertised yesterday, Marina was too/did too, even earlier. |
|  | different | When Deborah was excited by the flat that was advertised by the agency yesterday, Marina was too/did too, even earlier. |
| 113 | same | Whenever Rosie cleaned the flat that the students rented last year, Emma did too/was too, rather quickly. |
|  | different | Whenever Rosie cleaned the flat that was rented by the students last year, Emma did too/was too, rather quickly. |
| 114 | same | Whenever Hilary was apprehensive about the business that was blacklisted by the newspaper recently, Lindsey was too/did too, without fail. |
|  | different | Whenever Hilary was apprehensive about the business that the newspaper blacklisted recently, Lindsey was too/did too, without fail. |
| 115 | same | Because Vicky boarded the train that the inspector checked at the platform earlier, Katie did too/was too, without hesitation. |
|  | different | Because Vicky boarded the train that was checked by the inspector at the platform earlier, Katie did too/was too, without hesitation. |
| 116 | same | Because Juliana was caught by the camera that was checked by policemen sometime earlier, Angela was too/did too, yet again. |
|  | different | Because Juliana was caught by the camera that policemen checked sometime earlier, Angela was too/did too, yet again. |
| 117 | same | Since Harry passed the test that the teacher marked yesterday, Jamie did too/was too, as usual. |
|  | different | Since Harry passed the test that was marked by the teacher yesterday, Jamie did too/was too, as usual. |
| 118 | same | Since Darius was unsure about the suit that was on the mannequin in the window, Henry was too/did too, yet again. |
|  | different | Since Darius was unsure about the suit that the mannequin had on in the window, Henry was too/did too, yet again. |
| 119 | same | Because Lana drove the car that the mechanic fixed this morning, Daniel did too/was too, ignoring warnings. |
|  | different | Because Lana drove the car that was fixed by the mechanic this morning, Daniel did too/was too, ignoring warnings. |
| 120 | same | Because Alexis was excited about the job that was advertised by the hotel in newspapers, Christopher was too/did too, so much. |
|  | different | Because Alexis was excited about the job that the hotel advertised in newspapers, Christopher was too/did too, so much. |
| 121 | same | Whenever Milly sang the song that the band performed last summer, Jacky did too/was too, quite happily. |
|  | different | Whenever Milly sang the song that was performed by the band last summer, Jacky did too/was too, quite happily. |
| 122 | same | Whenever Susan was worried about the traffic that was caused by the lorries downtown, Alan was too/did too, so they cycled. |
|  | different | Whenever Susan was worried about the lorries that caused traffic downtown, Alan was too/did too, so they cycled. |
| 123 | same | Since Emily hung the washing that the maid finished last night, Jessica did too/was too, right outside. |
|  | different | Since Emily hung the washing that was finished by the maid last night, Jessica did too/was too, right outside. |
| 124 | same | Since Megan was annoyed about cars that were parked by the neighbours outside, Frances was too/did too, while complaining. |
|  | different | Since Megan was annoyed about cars that the neighbours parked outside, Frances was too/did too, while complaining. |
| 125 | same | Whenever Jenny cooked a meal that the neighbours enjoyed at dinner, Megan did too/was too, every time. |
|  | different | Whenever Jenny cooked a meal that was enjoyed by the neighbours at dinner, Megan did too/was too, every time. |
| 126 | same | Whenever Martha was harassed by the wasps that were spotted by the gardener last week, Lucy was too/did too, right away. |
|  | different | Whenever Martha was harassed by the wasps that the gardener spotted last week, Lucy was too/did too, right away. |
| 127 | same | Because Fiona finished the wine that her husband bought last Christmas, Jenny did too/was too, while dining. |
|  | different | Because Fiona finished the wine that was bought by her husband last Christmas, Jenny did too/was too, while dining. |
| 128 | same | Because Annette was splashed by the dog that was walked by the boy during showers, Irene was too/did too, right away. |
|  | different | Because Annette was splashed by the dog that the boy walked during showers, Jenny was too/did too, right away. |
| 129 | same | Since Hannah gate-crashed the party that the magazine organised last weekend, Louise did too/was too, wearing red. |
|  | different | Since Hannah gate-crashed the party that was organised by the magazine last weekend, Louise did too/was too, wearing red. |
| 130 | same | Since Audra was concerned about the deadline that was assigned by the lecturer this morning, Erica was too/did too, surprisingly so. |
|  | different | Since Audra was concerned about the deadline that the lecturer assigned this morning, Erica was too/did too, surprisingly so. |
| 131 | same | Because Megan booked the holiday that was recommended by the brochure last week, Vicky did too/was too, while it was cheap. |
|  | different | Because Megan booked the holiday that the brochure recommended last week, Vicky did too/was too, while it was cheap. |
| 132 | same | Because Amanda was approached by the scout that was employed by headhunters in London, Wilma was too/did too, to model. |
|  | different | Because Amanda was approached by the scout that headhunters employed in London, Wilma was too/did too, to model. |
| 133 | same | As Graham applied for the position that the boss advertised on Tuesday, Troy did too/was too, rather competitively. |
|  | different | As Graham applied for the position that was advertised by the boss on Tuesday, Troy did too/was too, rather competitively. |
| 134 | same | As David was applying for the job that was advertised by the consultant online, Jed was too/did too, as always. |
|  | different | As David was applying for the job that the consultant advertised online, Jed was too/did too, as always. |
| 135 | same | Since Margaret won a place that the school offered in Oxford, Kerry did too/was too, somewhat unexpectedly. |
|  | different | Since Margaret won a place that was offered by the school in Oxford, Kerry did too/was too, somewhat unexpectedly. |
| 136 | same | Since Tina was considering an MSc that was offered by the university last year, Katelyn was too/did too, disregarding expenses. |
|  | different | Since Tina was considering an MSc that the university offered last year, Katelyn was too/did too, disregarding expenses. |
| 137 | same | As Nora saw the band that hipsters followed ages ago, Mary did too/was too, just now. |
|  | different | As Nora saw the band that was followed by hipsters ages ago, Mary did too/was too, just now. |
| 138 | same | While Kitty was a fan of the comedian that was praised by critics ages ago, Pat was too/did too, but secretly. |
|  | different | While Kitty was a fan of the comedian that critics praised ages ago, Pat was too/did too, but secretly. |
| 139 | same | Because Charlie volunteered at the shelter that the council had opened nearby, James did too/was too, that evening. |
|  | different | Because Charlie volunteered at the shelter that was opened by the council nearby, James did too/was too, that evening. |
| 140 | same | Because Stephen was obliged to visit the gallery that was opened by his mother lately, Ryan was too/did too, as expected. |
|  | different | Because Stephen was obliged to visit the gallery that his mother opened lately, Ryan was too/did too, as expected. |
| 141 | same | As Ellie bought the toys that the family picked at Christmas, Gregory did too/was too, soon after. |
|  | different | As Ellie bought the toys that was picked by the family at Christmas, Gregory did too/was too, soon after. |
| 142 | same | As Carrie was asked about figures that were presented by the company eventually, Jerome was too/did too, soon after. |
|  | different | As Carrie was asked about figures that the company presented eventually, Jerome was too/did too, soon after. |
| 143 | same | Since Jasmine joined the trip that the school organised in the holidays, Hillary did too/was too, without hesitation. |
|  | different | Since Jasmine joined the trip that was organised by the school in the holidays, Hillary did too/was too, without hesitation. |
| 144 | same | Since Cindy was allowed a treat that was provided by the mother yesterday, Joanna was too/did too, once more. |
|  | different | Since Cindy was allowed a treat that the mother provided yesterday, Joanna was too/did too, once more. |
| 145 | same | When Julia played with the dolls that grandma had given as a surprise yesterday, Nina did too/was too, with pleasure. |
|  | different | When Julia played with the dolls that were given by grandma as a surprise yesterday, Nina did too/was too, with pleasure. |
| 146 | same | When Ivy was tired the dolls that were given by grandma yesterday, Zoe was too/did too, not surprisingly. |
|  | different | When Ivy was tired the dolls that grandma had given her yesterday, Zoe was too/did too, not surprisingly. |
| 147 | same | As Beatrice ordered the necklace that the stylist endorsed on TV, Gabby did too/was too, without hesitation. |
|  | different | As Beatrice ordered the necklace that was endorsed by the stylist on TV, Gabby did too/was too, without hesitation. |
| 148 | same | After Charlotte was excited about the gift that was delivered by friends Christmas morning, Kelly was too/did too, later on. |
|  | different | After Charlotte was excited about the gift that friends delivered Christmas morning, Kelly was too/did too, later on. |
| 149 | same | Since Paula ignored the bills that the council sent every week, Lauren did too/was too, until now. |
|  | different | Since Paula ignored the bills that were sent by the council every week, Lauren did too/was too, until now. |
| 150 | same | Since Michelle was hit by the door that was pushed by children in the lobby, Brooke was too/did too, pretty badly. |
|  | different | Since Michelle was hit by the door that children pushed in the lobby, Brooke was too/did too, pretty badly. |
| 151 | same | Because Will ignored the sound that the floorboards made in the hallway, Tom did too/was too, though hesitantly. |
|  | different | Because Will ignored the sound that was made by the floorboards in the hallway, Tom did too/was too, though hesitantly. |
| 152 | same | Because Rob was offended by the graffiti that was painted by hoodlums on the wall, Kirk was too/did too, surprisingly so. |
|  | different | Because Rob was offended by the graffiti that hoodlums painted on the wall, Kirk was too/did too, surprisingly so. |
| 153 | same | Since Joe wanted the toy that the TV programme advertised daily, Jack did too/was too, for fun. |
|  | different | Since Joe wanted the toy that was advertised by the TV programme daily, Jack did too/was too, for fun. |
| 154 | same | Since Andrew was encouraged by the feedback that the interviewer reported via email, Ivor was too/did too, at once. |
|  | different | Since Andrew was encouraged by the feedback that was reported by the interviewer via email, Ivor was too/did too, at once. |
| 155 | same | Because Andrea joined the gym that the personal trainer found online, Rosie did too/was too, with enthusiasm. |
|  | different | Because Andrea joined the gym that was found by the personal trainer online, Rosie did too/was too, with enthusiasm. |
| 156 | same | Because Natalie was terrified about the flight that was booked by her boss recently, Judy was too/did too, and cried. |
|  | different | Because Natalie was terrified about the flight that her boss booked recently, Judy was too/did too, and cried. |

Critical sentences for Experiment 2.

| Item | Attractor plausibility | Sentence |
| --- | --- | --- |
| 1 | implausible | Because John saw the film that was reviewed by the critics yesterday, Bill did too/was too, just now. |
|  | plausible | Because John saw the film that was seen by the critics yesterday, Bill did too/was too, just now. |
| 2 | implausible | Because Jim was excited by the book that critics summarized in the ad, Rob was too/did too, after all. |
|  | plausible | Because Jim was excited by the book that critics praised in the ad, Rob was too/did too, after all. |
| 3 | implausible | Because Tom liked the cake that was prepared by the chef earlier, Jack did too/was too, quite unexpectedly. |
|  | plausible | Because Tom liked the cake that was served by the chef earlier, Jack did too/was too, quite unexpectedly. |
| 4 | implausible | Because Will was tempted by the cake that the café displayed last week, Carl was too/did too, after lunch. |
|  | plausible | Because Will was tempted by the cake that the café introduced last week, Carl was too/did too, after lunch. |
| 5 | implausible | Since Sally bought the perfume that was worn by the actress on TV, Holly did too/was too, on sale. |
|  | plausible | Since Sally bought the perfume that was recognised by the actress on TV, Holly did too/was too, on sale. |
| 6 | implausible | Since Sue was pleased by the dresses that the tailor altered yesterday, Eva was too/did too, as expected. |
|  | plausible | Since Sue was pleased by the dresses that the tailor chose yesterday, Eva was too/did too, as expected. |
| 7 | implausible | After Lee received the essay that was marked by the lecturer last week, Seamus did too/was too, in class. |
|  | plausible | After Lee received the essay that was criticised by the lecturer last week, Seamus did too/was too, in class. |
| 8 | implausible | After Liam was praised for work that the invigilator marked yesterday, Lewis was too/did too, on Friday. |
|  | plausible | After Liam was praised for work that the invigilator inspected yesterday, Lewis was too/did too, on Friday. |
| 9 | implausible | Whenever Toni read novels that were endorsed by the book club in the past week, Anne did too/was too, without complaining. |
|  | plausible | Whenever Toni read novels that were collected by the book club in the past week, Anne did too/was too, without complaining. |
| 10 | implausible | Whenever Tamsin was stressed by the party that the employees planned at work, Hannah was too/did too, despite tranquilizers. |
|  | plausible | Whenever Tamsin was stressed by the party that the employees mentioned at work, Hannah was too/did too, despite tranquilizers. |
| 11 | implausible | Since Sandy bought the tickets that were advertised by the theatre in the magazine, Bill did too/was too, rather reluctantly. |
|  | plausible | Since Sandy bought the tickets that were put forward by the theatre in the magazine, Bill did too/was too, rather reluctantly. |
| 12 | implausible | Since Chloe was scared by the knife that the butcher practiced with that night, Sam was too/did too, quite rightly. |
|  | plausible | Since Chloe was scared by the knife that the butcher sharpened that night, Sam was too/did too, quite rightly. |
| 13 | implausible | Because Georgia accepted the flowers that were arranged by the benefactor in the shop, Hannah did too/was too, somewhat reluctantly. |
|  | plausible | Because Georgia accepted the flowers that were spotted by the benefactor in the shop, Hannah did too/was too, somewhat reluctantly. |
| 14 | implausible | Because Gina was unimpressed by the cocktail that the bartender had mixed with care, Erik was too/did too, as always. |
|  | plausible | Because Gina was unimpressed by the cocktail that the bartender had served with care, Erik was too/did too, as always. |
| 15 | implausible | Because Philip rejected the offer that was proposed by the negotiator in the meeting, Danny did too/was too, that afternoon. |
|  | plausible | Because Philip rejected the offer that was seconded by the negotiator in the meeting, Danny did too/was too, that afternoon. |
| 16 | implausible | Because Arthur was ridiculed by the article that the blogger wrote online, Anna was too/did too, sadly enough. |
|  | plausible | Because Arthur was ridiculed by the article that the blogger followed online, Anna was too/did too, sadly enough. |
| 17 | implausible | As Jessica evaluated the transcripts that were submitted by the student to the journal, Rachel did too/was too, to double-check. |
|  | plausible | As Jessica evaluated the transcripts that were suggested by the student to the journal, Rachel did too/was too, to double-check. |
| 18 | implausible | Because Jane was interested by the car that the woman advertised online, Ellen was too/did too, causing problems. |
|  | plausible | Because Jane was interested by the car that the woman spotted online, Ellen was too/did too, causing problems. |
| 19 | implausible | Because Pete liked the song that was played by the radio presenter this morning, Todd did too/was too, quite surprisingly. |
|  | plausible | Because Pete liked the song that was promoted by the radio presenter this morning, Todd did too/was too, quite surprisingly. |
| 20 | implausible | Because Simon was shocked by the painting that the artist finished earlier, Richard was too/did too, rather clearly. |
|  | plausible | Because Simon was shocked by the painting that the artist nominated earlier, Richard was too/did too, rather clearly. |
| 21 | implausible | Since Leah completed the crossword that was published by the newspaper yesterday, Cassie did too/was too, during lunch. |
|  | plausible | Since Leah completed the crossword that was selected by the newspaper yesterday, Cassie did too/was too, during lunch. |
| 22 | implausible | Since Amy was upset by the trap that the farmer set last night, Kelly was too/did too, quite understandably. |
|  | plausible | Since Amy was upset by the trap that the farmer saw last night, Kelly was too/did too, quite understandably. |
| 23 | implausible | As Cleo ran the race that was organised by the trainer last summer, Emma did too/was too, somewhat reluctantly. |
|  | plausible | As Cleo ran the race that was known by the trainer last summer, Emma did too/was too, somewhat reluctantly. |
| 24 | implausible | As Lena was seen by the camera that policemen fitted in the cabinet, Laura was too/did too, moments later. |
|  | plausible | As Lena was seen by the camera that policemen noticed in the cabinet, Laura was too/did too, moments later. |
| 25 | implausible | As Beth phoned the company that was used by the neighbours over lunch, Anna did too/was too, right away. |
|  | plausible | As Beth phoned the company that was recommended by the neighbours over lunch, Anna did too/was too, right away. |
| 26 | implausible | As Camille was intrigued by the story that the actor created yesterday, Mia was too/did too, at once. |
|  | plausible | As Camille was intrigued by the story that the actor told yesterday, Mia was too/did too, at once. |
| 27 | implausible | Because Chelsea wrapped the presents that were bought by the children on Saturday, Jo did too/was too, very quickly. |
|  | plausible | Because Chelsea wrapped the presents that were found by the children on Saturday, Jo did too/was too, very quickly. |
| 28 | implausible | Because Gemma was invited to the hotel that the journalist stayed at that night, Holly was too/did too, months later. |
|  | plausible | Because Gemma was invited to the hotel that the journalist targeted that night, Holly was too/did too, months later. |
| 29 | implausible | Since Alexa stole the sweets that were displayed by the shopkeeper at the checkout, Lisbeth did too/was too, after all. |
|  | plausible | Since Alexa stole the sweets that were left by the shopkeeper at the checkout, Lisbeth did too/was too, after all. |
| 30 | implausible | Since Marjory was annoyed by the ball that the youths threw in the street, Catherine was too/did too, but less. |
|  | plausible | Since Marjory was annoyed by the ball that the youths kicked in the street, Catherine was too/did too, but less. |
| 31 | implausible | Because Joe entered the competition that was created by the teacher yesterday, Richard did too/was too, but reluctantly. |
|  | plausible | Because Joe entered the competition that was recommended by the teacher yesterday, Richard did too/was too, but reluctantly. |
| 32 | implausible | Because Jamie was disliked by the team that businessmen disbanded years ago, Felix was too/did too, even more. |
|  | plausible | Because Jamie was disliked by the team that businessmen sponsored years ago, Felix was too/did too, even more. |
| 33 | implausible | Since Jen ate the curry that was prepared by the vendor on the street, Hannah did too/was too, as dinner. |
|  | plausible | Since Jen ate the curry that was offered by the vendor on the street, Hannah did too/was too, as dinner. |
| 34 | implausible | Since Sally was frightened by the costume that the clown wore that evening, Nina was too/did too, quite understandably. |
|  | plausible | Since Sally was frightened by the costume that the clown chose that evening, Nina was too/did too, quite understandably. |
| 35 | implausible | As Mike attended the meeting that was scheduled by the company last week, Ryan did too/was too, as always. |
|  | plausible | As Mike attended the meeting that was called by the company last week, Ryan did too/was too, as always. |
| 36 | implausible | When Bill was disgusted by the tooth that the dentist removed recently, John was too/did too, at once. |
|  | plausible | When Bill was disgusted by the tooth that the dentist examined recently, John was too/did too, at once. |
| 37 | implausible | Because Harriet wore the uniform that was ironed by the seamstress yesterday, Felicity did too/was too, after deliberation. |
|  | plausible | Because Harriet wore the uniform that was complimented by the seamstress yesterday, Felicity did too/was too, after deliberation. |
| 38 | implausible | Because Helen was alarmed by a siren that the ambulance sounded outside, Felicity was too/did too, moments later. |
|  | plausible | Because Helen was alarmed by a siren that the ambulance relied on outside, Felicity was too/did too, moments later. |
| 39 | implausible | While Jeremy drew the sunset that was captured by the photograph last night, Declan did too/was too, quite beautifully. |
|  | plausible | While Jeremy drew the sunset that was featured in the photograph last night, Declan did too/was too, quite beautifully. |
| 40 | implausible | While Richard was licked by the dog that the vet fed today, Declan was too/did too, a lot. |
|  | plausible | While Richard was licked by the dog that the vet sponsored today, Declan was too/did too, a lot. |
| 41 | implausible | Since Keira followed the car that was owned by the mayor on Saturday, Hannah did too/was too, at night. |
|  | plausible | Since Keira followed the car that was liked by the mayor on Saturday, Hannah did too/was too, at night. |
| 42 | implausible | Since Elizabeth was enticed by the dress that the celebrity wore this month, Hannah was too/did too, quite understandably. |
|  | plausible | Since Elizabeth was enticed by the dress that the celebrity promoted this month, Hannah was too/did too, quite understandably. |
| 43 | implausible | Because Paul liked the training course that was vetoed by his boss this year, Neil did too/was too, in agreement. |
|  | plausible | Because Paul liked the training course that was recommended by his boss this year, Neil did too/was too, in agreement. |
| 44 | implausible | Because Jake was woken by the curses made by the people in the street, Neil was too/did too, that morning. |
|  | plausible | Because Jake was woken by the curses called by the people in the street, Neil was too/did too, that morning. |
| 45 | implausible | Because Fred denounced the article that was triaged by the editor yesterday, Murray did too/was too, at last. |
|  | plausible | Because Fred denounced the article that was insulted by the editor yesterday, Murray did too/was too, at last. |
| 46 | implausible | Because George was angered by the article that the journalist wrote online, Abe was too/did too, very predictably. |
|  | plausible | Because George was angered by the article that the journalist lambasted online, Abe was too/did too, very predictably. |
| 47 | implausible | Because Chloe distrusted the marketing strategy that was used by the shop on many occasions, Sarah did too/was too, without question. |
|  | plausible | Because Chloe distrusted the marketing strategy that was employed by the shop on many occasions, Sarah did too/was too, without question. |
| 48 | implausible | Because Diana was interested by the formula that the scientist centrifuged regularly, Jessica was too/did too, later on. |
|  | plausible | Because Diana was interested by the formula that the scientist described regularly, Jessica was too/did too, later on. |
| 49 | implausible | Since Katherine went to the art fair that was inaugurated by royalty yesterday, Lynn did too/was too, on Monday. |
|  | plausible | Since Katherine went to the art fair that was commended by royalty yesterday, Lynn did too/was too, on Monday. |
| 50 | implausible | Since Emily was interested by visiting the exhibition that the artist sold in frequently, Brenda was too/did too, a bit. |
|  | plausible | Since Emily was interested by visiting the exhibition that the artist mentioned frequently, Brenda was too/did too, a bit. |
| 51 | implausible | As Dylan played the game that was made up by his friend at lunchtime, Will did too/was too, on Thursday. |
|  | plausible | As Dylan played the game that was mimicked by his friend at lunchtime, Will did too/was too, on Thursday. |
| 52 | implausible | As Avery was delighted by the concert that the conductor had organised on Friday, Jake was too/did too, pretty much. |
|  | plausible | As Avery was delighted by the concert that the conductor had permitted on Friday, Jake was too/did too, pretty much. |
| 53 | implausible | Because Kim enjoyed the tasting that was prepared by the vineyard yesterday, Chris did too/was too, as usual. |
|  | plausible | Because Kim enjoyed the tasting that was hosted by the vineyard yesterday, Chris did too/was too, as usual. |
| 54 | implausible | Because Nina was disgruntled by the gift that her mum purchased for them earlier, Mike was too/did too, as expected. |
|  | plausible | Because Nina was disgruntled by the gift that her mum complained to them about earlier, Mike was too/did too, as expected. |
| 55 | implausible | While Tim read the book that was used by the class for the semester, Alice did too/was too, yesterday evening. |
|  | plausible | While Tim read the book that was praised by the class for the semester, Alice did too/was too, yesterday evening. |
| 56 | implausible | While Alexander was annoyed by the project that the examiners planned as coursework, Matilde was too/did too, even more. |
|  | plausible | While Alexander was annoyed by the project that the examiners appreciated as coursework, Matilde was too/did too, even more. |
| 57 | implausible | Since Harry stole the whiskey that was opened by his dad recently, Rachel did too/was too, on Saturday. |
|  | plausible | Since Harry stole the whiskey that was found by his dad recently, Rachel did too/was too, on Saturday. |
| 58 | implausible | Since Colm was envious of the car that his stepdad bought recently, Isabelle was too/did too, pretty much. |
|  | plausible | Since Colm was envious of the car that his stepdad admired recently, Isabelle was too/did too, pretty much. |
| 59 | implausible | Because Nikki listened to the song that was composed by her brother on the motorway, Rosie did too/was too, after all. |
|  | plausible | Because Nikki listened to the song that was recommended by her brother on the motorway, Rosie did too/was too, after all. |
| 60 | implausible | Because Terry was disturbed by the music that bloggers posted yesterday, Pamela was too/did too, as always. |
|  | plausible | Because Terry was disturbed by the music that bloggers suggested yesterday, Pamela was too/did too, as always. |
| 61 | implausible | As Maria chose the most expensive wine that was stocked by the restaurant that night, Ben did too/was too, as always |
|  | plausible | As Maria chose the most expensive wine that was served by the restaurant that night, Ben did too/was too, as always |
| 62 | implausible | As Judith was impressed by the cheese that the restaurant imported recently, Johnny was too/did too, rather unexpectedly. |
|  | plausible | As Judith was impressed by the cheese that the restaurant served recently, Johnny was too/did too, rather unexpectedly. |
| 63 | implausible | While Claire mentioned vaccinations that were purchased by the family on holiday, Carolyn did too/was too, somewhat later. |
|  | plausible | While Claire mentioned vaccinations that were needed by the family on holiday, Carolyn did too/was too, somewhat later. |
| 64 | implausible | While Tessa was delighted by the clothes that her daughter got on holiday, Ruby was too/did too, straight away. |
|  | plausible | While Tessa was delighted by the clothes that her daughter took on holiday, Ruby was too/did too, straight away. |
| 65 | implausible | Since Steven ate the cake that was baked by his daughter for his birthday party, Taylor did too/was too, when offered. |
|  | plausible | Since Steven ate the cake that was offered by his daughter for his birthday party, Taylor did too/was too, when offered. |
| 66 | implausible | Since Rene was obliged to eat the meal that his son had cooked recently, Dan was too/did too, when offered. |
|  | plausible | Since Rene was obliged to eat the meal that his son had helped with recently, Dan was too/did too, when offered. |
| 67 | implausible | Because Martha decided to visit the café that was advertised by the magazine often, Becky did too/was too, that day. |
|  | plausible | Because Martha decided to visit the café that was interviewed by the magazine often, Becky did too/was too, that day. |
| 68 | implausible | Because Vicki was seduced by the deal that the magazine advertised often, Norma was too/did too, as usual. |
|  | plausible | Because Vicki was seduced by the deal that the magazine spotted often, Norma was too/did too, as usual. |
| 69 | implausible | As Joe gave money to the charity that was run by the school occasionally, Sally did too/was too, to help. |
|  | plausible | As Joe gave money to the charity that was supported by the school occasionally, Sally did too/was too, to help. |
| 70 | implausible | As Armin was drafted by companies that alumni had founded in recent years, Lorelei was too/did too, surprisingly enough. |
|  | plausible | As Armin was drafted by companies that alumni associated with in recent years, Lorelei was too/did too, surprisingly enough. |
| 71 | implausible | Since Tina liked the training machine that was purchased by the team in the new year, Clara did too/was too, after all. |
|  | plausible | Since Tina liked the training machine that was introduced by the team in the new year, Clara did too/was too, after all. |
| 72 | implausible | Since Nicole was affected by the flu that the doctor diagnosed last month, Mary was too/did too, but worse. |
|  | plausible | Since Nicole was affected by the flu that the doctor cured last month, Mary was too/did too, but worse. |
| 73 | implausible | While Andy got ready for the party that was planned on Facebook recently, Edward did too/was too, rather reluctantly. |
|  | plausible | While Andy got ready for the party that was popular on Facebook recently, Edward did too/was too, rather reluctantly. |
| 74 | implausible | While Michael was calculating the sum that the accountants sorted out annually, Luke was too/did too, very quickly. |
|  | plausible | While Michael was calculating the sum that the accountants complained about annually, Luke was too/did too, very quickly. |
| 75 | implausible | When Kelly entered the marathon that was held by her town again, Ollie did too/was too, as usual. |
|  | plausible | When Kelly entered the marathon that was promoted by her town again, Ollie did too/was too, as usual. |
| 76 | implausible | When Cathy was excited by the rally that the town held again, Brian was too/did too, not surprisingly. |
|  | plausible | When Cathy was excited by the rally that the town supported again, Brian was too/did too, not surprisingly. |
| 77 | implausible | As Emma went to the exercise class that was organised by the doctor yesterday, Carina did too/was too, as company. |
|  | plausible | As Emma went to the exercise class that was suggested by the doctor yesterday, Carina did too/was too, as company. |
| 78 | implausible | As Rose was excited by the physiotherapy that the doctor invented recently, Gloria was too/did too, as expected. |
|  | plausible | As Rose was excited by the physiotherapy that the doctor mentioned recently, Gloria was too/did too, as expected. |
| 79 | implausible | Because Matt got a meal that was sold at the take-away that night, Andrea did too/was too, rather quickly. |
|  | plausible | Because Matt got a meal that was famous at the take-away that night, Andrea did too/was too, rather quickly. |
| 80 | implausible | Because Robert was reassured by the contract that his client signed that day, Kelly was too/did too, minutes later. |
|  | plausible | Because Robert was reassured by the contract that his client corrected that day, Kelly was too/did too, minutes later. |
| 81 | implausible | Because Peter went to the only comedy that was listed by the cinema that week, Simon did too/was too, quite happily. |
|  | plausible | Because Peter went to the only comedy that was seen by the cinema that week, Simon did too/was too, quite happily. |
| 82 | implausible | Because Ian was impressed by the comedy that the cinemas released lately, Noah was too/did too, at once. |
|  | plausible | Because Ian was impressed by the comedy that the cinemas showed lately, Noah was too/did too, at once. |
| 83 | implausible | As Daisy criticised the game that was received by her son as present yesterday, Hannah did too/was too, very harshly. |
|  | plausible | As Daisy criticised the game that was favoured by her son as present yesterday, Hannah did too/was too, very harshly. |
| 84 | implausible | As Caroline was dismayed by the toy gun that her son bought yesterday, Tracy was too/did too, last night. |
|  | plausible | As Caroline was dismayed by the toy gun that her son cherished yesterday, Tracy was too/did too, last night. |
| 85 | implausible | While Max listened to the album that was released by the band recently, Tim did too/was too, to check. |
|  | plausible | While Max listened to the album that was preferred by the band recently, Tim did too/was too, to check. |
| 86 | implausible | While Barry was queuing for the gadget that Tesco discounted recently, Maxim was too/did too, quite excitedly. |
|  | plausible | While Barry was queuing for the gadget that Tesco promoted recently, Maxim was too/did too, quite excitedly. |
| 87 | implausible | Since Siobhan admitted the flaw that was pointed out by the office last week, Duncan did too/was too, rather honestly |
|  | plausible | Since Siobhan admitted the flaw that was found by the office last week, Duncan did too/was too, rather honestly |
| 88 | implausible | Since Cyrus was upset by the policy that the office changed last week, Bradley was too/did too, very much. |
|  | plausible | Since Cyrus was upset by the policy that the office ignored last week, Bradley was too/did too, very much. |
| 89 | implausible | When Rebecca went to the house that was refurbished by her grandma during summer, Claudia did too/was too, yesterday afternoon. |
|  | plausible | When Rebecca went to the house that was looked after by her grandma during summer, Claudia did too/was too, yesterday afternoon. |
| 90 | implausible | When Penny was bothered by her dog who barked often, Melinda was too/did too, not surprisingly. |
|  | plausible | When Penny was bothered by her dog who cried often, Melinda was too/did too, not surprisingly. |
| 91 | implausible | Since Anna chose the cake that was finished by the chef for dessert, Kerry did too/was too, after deliberating. |
|  | plausible | Since Anna chose the cake that was offered by the chef for dessert, Kerry did too/was too, after deliberating. |
| 92 | implausible | Since Salome was intrigued by the story that the refugee wrote in the report, Mark was too/did too, rather surprisingly. |
|  | plausible | Since Salome was intrigued by the story that the refugee told in the report, Mark was too/did too, rather surprisingly. |
| 93 | implausible | As Rob took part in the protest that was run by the society for charity, Freya did too/was too, quite bravely. |
|  | plausible | As Rob took part in the protest that was called by the society for charity, Freya did too/was too, quite bravely. |
| 94 | implausible | As Ronnie was disgusted by the scheme that charities ran recently, Heather was too/did too, although less. |
|  | plausible | As Ronnie was disgusted by the scheme that charities accepted recently, Heather was too/did too, although less. |
| 95 | implausible | Because Tyler climbed the glacier that was attempted by experts this year, Gregg did too/was too, very willingly. |
|  | plausible | Because Tyler climbed the glacier that was investigated by experts this year, Gregg did too/was too, very willingly. |
| 96 | implausible | Because Kenneth was astounded by the mountain that experts explored last year, Patrick was too/did too, of course. |
|  | plausible | Because Kenneth was astounded by the mountain that experts feared last year, Patrick was too/did too, of course. |
| 97 | implausible | While Hamish cleaned the flat that was inventoried by the landlords monthly, Ally did too/was too, after work. |
|  | plausible | While Hamish cleaned the flat that was visited by the landlords monthly, Ally did too/was too, after work. |
| 98 | implausible | Since Kevin was being prepared for the inspection that managers ran monthly, Jordan was too/did too, rather slowly. |
|  | plausible | Since Kevin was being prepared for the inspection that managers required monthly, Jordan was too/did too, rather slowly. |
| 99 | implausible | As Terry made biscuits that were eaten by the neighbours again, Eva did too/was too, once again. |
|  | plausible | As Terry made biscuits that were loved by the neighbours again, Eva did too/was too, once again. |
| 100 | implausible | As Susie was enlisted for the event that villagers organised again, Roxanne was too/did too, without choice. |
|  | plausible | As Susie was enlisted for the event that villagers directed again, Roxanne was too/did too, without choice. |
| 101 | implausible | When Kyle considered the computer that was analysed in the report lately, Nick did too/was too, for purchase. |
|  | plausible | When Kyle considered the computer that was recommended in the report lately, Nick did too/was too, for purchase. |
| 102 | implausible | When Jerry was spammed by the website that the hacker accessed repeatedly, Ethan was too/did too, very shockingly. |
|  | plausible | When Jerry was spammed by the website that the hacker tricked repeatedly, Ethan was too/did too, very shockingly. |
| 103 | implausible | Because Harriet went to the protest that was planned by the students yesterday, Holly did too/was too, to help. |
|  | plausible | Because Harriet went to the protest that was followed by the students yesterday, Holly did too/was too, to help. |
| 104 | implausible | Because Melanie was excited by the rally that protestors organised this weekend, Jean was too/did too, very much. |
|  | plausible | Because Melanie was excited by the rally that protestors joined this weekend, Jean was too/did too, very much. |
| 105 | implausible | While Amy washed the clothes that were worn by the models on the catwalk yesterday, Vicky did too/was too, feeling tired. |
|  | plausible | While Amy washed the clothes that were selected by the models on the catwalk yesterday, Vicky did too/was too, feeling tired. |
| 106 | implausible | While Cecily was stressed by the essay that the teacher set last week, Scarlett was too/did too, quite deeply. |
|  | plausible | While Cecily was stressed by the essay that the teacher assigned last week, Scarlett was too/did too, quite deeply. |
| 107 | implausible | Because Becky drank the tea that was brewed by the waitress minutes earlier, Erin did too/was too, to relax. |
|  | plausible | Because Becky drank the tea that was served by the waitress minutes earlier, Erin did too/was too, to relax. |
| 108 | implausible | Because Shauna was mocked by the mouse that the shopkeeper trapped last week, Nancy was too/did too, feeling terrified. |
|  | plausible | Because Shauna was mocked by the mouse that the shopkeeper identified last week, Nancy was too/did too, feeling terrified. |
| 109 | implausible | Since Christine ate the curry that was cooked by the chef yesterday, Sophie did too/was too, for lunch. |
|  | plausible | Since Christine ate the curry that was introduced by the chef yesterday, Sophie did too/was too, for lunch. |
| 110 | implausible | Since Paul was delayed by the flight that the pilot navigated in blizzard conditions, John was too/did too, quite obviously. |
|  | plausible | Since Paul was delayed by the flight that the pilot led in blizzard conditions, John was too/did too, quite obviously. |
| 111 | implausible | When Beth wanted the job that was highlighted by the company in accounting, Anna did too/was too, very desperately. |
|  | plausible | When Beth wanted the job that was offered by the company in accounting, Anna did too/was too, very desperately. |
| 112 | implausible | When Deborah was excited by the flat that the agency advertised yesterday, Marina was too/did too, very much. |
|  | plausible | When Deborah was excited by the flat that the agency mentioned yesterday, Marina was too/did too, very much. |
| 113 | implausible | Whenever Rosie cleaned the castle that was maintained by the city since last year, Emma did too/was too, very elaborately. |
|  | plausible | Whenever Rosie cleaned the castle that was protected by the city since last year, Emma did too/was too, very elaborately. |
| 114 | implausible | Whenever Hilary was apprehensive about the business that the newspaper blacklisted recently, Lindsey was too/did too, very understandably. |
|  | plausible | Whenever Hilary was apprehensive about the business that the newspaper blamed recently, Lindsey was too/did too, very understandably. |
| 115 | implausible | Because Vicky boarded the train that was regulated by the inspector at the platform earlier, Katie did too/was too, in haste. |
|  | plausible | Because Vicky boarded the train that was stopped by the inspector at the platform earlier, Katie did too/was too, in haste. |
| 116 | implausible | Because Juliana was caught by the camera that policemen detached sometime earlier, Angela was too/did too, at midnight. |
|  | plausible | Because Juliana was caught by the camera that policemen located sometime earlier, Angela was too/did too, at midnight. |
| 117 | implausible | Since Harry passed the test that was marked by the teacher yesterday, Jamie did too/was too, quite naturally. |
|  | plausible | Since Harry passed the test that was presented by the teacher yesterday, Jamie did too/was too, quite naturally. |
| 118 | implausible | Since Darius was unsure about the suit that the mannequin exhibited in the window, Henry was too/did too, a little. |
|  | plausible | Since Darius was unsure about the suit that the mannequin wore in the window, Henry was too/did too, a little. |
| 119 | implausible | Because Lana drove the car that was fixed by the mechanic this morning, Daniel did too/was too, for work. |
|  | plausible | Because Lana drove the car that was dealt with by the mechanic this morning, Daniel did too/was too, for work. |
| 120 | implausible | Because Alexis was excited by the job that the hotel advertised in newspapers, Christopher was too/did too, so much. |
|  | plausible | Because Alexis was excited by the job that the hotel commented on in newspapers, Christopher was too/did too, so much. |
| 121 | implausible | Whenever Millie sang the song that was performed by the band last summer, Jacky did too/was too, very loudly. |
|  | plausible | Whenever Millie sang the song that was announced by the band last summer, Jacky did too/was too, very loudly. |
| 122 | implausible | Whenever Susan was bothered by the lorries that snarled up traffic downtown, Alan was too/did too, more badly. |
|  | plausible | Whenever Susan was bothered by the lorries that controlled traffic downtown, Alan was too/did too, more badly. |
| 123 | implausible | Since Emily hung up the washing that was finished by the maid last night, Jessica did too/was too, without moaning. |
|  | plausible | Since Emily hung up the washing that was hated by the maid last night, Jessica did too/was too, without moaning. |
| 124 | implausible | Since Megan was annoyed by cars that the neighbours parked outside, Frances was too/did too, every day. |
|  | plausible | Since Megan was annoyed by cars that the neighbours left outside, Frances was too/did too, every day. |
| 125 | implausible | Whenever Jenny cooked a meal that was enjoyed by the neighbours at dinner, Megan did too/was too, as always. |
|  | plausible | Whenever Jenny cooked a meal that was requested by the neighbours at dinner, Megan did too/was too, as always. |
| 126 | implausible | Whenever Martha was harassed by the wasps that the gardener eradicated last week, Lucy was too/did too, quite badly. |
|  | plausible | Whenever Martha was harassed by the wasps that the gardener found last week, Lucy was too/did too, quite badly. |
| 127 | implausible | Because Fiona finished the wine that was bought by her husband last Christmas, Jenny did too/was too, very happily. |
|  | plausible | Because Fiona finished the wine that was introduced by her husband last Christmas, Jenny did too/was too, very happily. |
| 128 | implausible | Because Annette was splashed by the dog that the boy walked during the rain shower, Jenny was too/did too, rather terribly. |
|  | plausible | Because Annette was splashed by the dog that the boy amused during the rain shower, Jenny was too/did too, rather terribly. |
| 129 | implausible | Since Hannah gate-crashed the party that was organised by the magazine last weekend, Louise did too/was too, following her. |
|  | plausible | Since Hannah gate-crashed the party that was featured by the magazine last weekend, Louise did too/was too, following her. |
| 130 | implausible | Since Audra was concerned about the deadline that the lecturer decided this morning, Erica was too/did too, slightly more. |
|  | plausible | Since Audra was concerned about the deadline that the lecturer assigned this morning, Erica was too/did too, slightly more. |
| 131 | implausible | Because Megan booked the holiday that was covered by the brochure last week, Vicky did too/was too, that night. |
|  | plausible | Because Megan booked the holiday that was described by the brochure last week, Vicky did too/was too, that night. |
| 132 | implausible | Because Amanda was approached by the institute that her relative ruled in London, Wilma was too/did too, rather inevitably. |
|  | plausible | Because Amanda was approached by the institute that her relative counted on in London, Wilma was too/did too, rather inevitably. |
| 133 | implausible | As Graham applied for the position that was advertised by the boss on Tuesday, Troy did too/was too, quite willingly. |
|  | plausible | As Graham applied for the position that was supervised by the boss on Tuesday, Troy did too/was too, quite willingly. |
| 134 | implausible | As David was interested by the job that the consultant advertised online, Jed was too/did too, for sure. |
|  | plausible | As David was interested by the job that the consultant overrated online, Jed was too/did too, for sure. |
| 135 | implausible | Since Margaret won a place that was established by the school in Oxford, Kerry did too/was too, as expected. |
|  | plausible | Since Margaret won a place that was offered by the school in Oxford, Kerry did too/was too, as expected. |
| 136 | implausible | Since Tina was interested in an MSc that the university launched last year, Katelyn was too/did too, with excitement. |
|  | plausible | Since Tina was interested in an MSc that the university funded last year, Katelyn was too/did too, with excitement. |
| 137 | implausible | As Nora saw the band that was formed by hipsters ages ago, Mary did too/was too, after dinner. |
|  | plausible | As Nora saw the band that was imitated by hipsters ages ago, Mary did too/was too, after dinner. |
| 138 | implausible | While Kitty was a fan of the comedy show that the group established ages ago, Pat was too/did too, for ages. |
|  | plausible | While Kitty was a fan of the comedy show that the group supported ages ago, Pat was too/did too, for ages. |
| 139 | implausible | Because Charlie volunteered at the shelter that was opened by the council nearby, James did too/was too, for fun. |
|  | plausible | Because Charlie volunteered at the shelter that was directed by the council nearby, James did too/was too, for fun. |
| 140 | implausible | Because Stephen was obliged to visit the gallery that his mother opened lately, Ryan was too/did too, on Sunday. |
|  | plausible | Because Stephen was obliged to visit the gallery that his mother visited lately, Ryan was too/did too, on Sunday. |
| 141 | implausible | As Ellie bought the toy that was listed by the family at Christmas, Carrie did too/was too, after checking. |
|  | plausible | As Ellie bought the toy that was chosen by the family at Christmas, Carrie did too/was too, after checking. |
| 142 | implausible | As Gregory was asked about figures that the company published eventually, Jerome was too/did too, in detail. |
|  | plausible | As Gregory was asked about figures that the company showed eventually, Jerome was too/did too, in detail. |
| 143 | implausible | Since Jasmine joined the trip that was organised by the school in the holidays, Hillary did too/was too, this year. |
|  | plausible | Since Jasmine joined the trip that was suggested by the school in the holidays, Hillary did too/was too, this year. |
| 144 | implausible | Since Cindy was given a treat that her mother bought yesterday, Joanna was too/did too, after school. |
|  | plausible | Since Cindy was given a treat that her mother allowed yesterday, Joanna was too/did too, after school. |
| 145 | implausible | When Julia played with the dolls that were made by her grandma as a surprise yesterday, Nina did too/was too, looking happy. |
|  | plausible | When Julia played with the dolls that were given by her grandma as a surprise yesterday, Nina did too/was too, looking happy. |
| 146 | implausible | When Ivy was delighted by the dolls that her grandma had fixed for her yesterday, Zoe was too/did too, straight after. |
|  | plausible | When Ivy was delighted by the dolls that her grandma had found for her yesterday, Zoe was too/did too, straight after. |
| 147 | implausible | As Beatrice ordered the necklace that was designed by the stylist on TV, Gabby did too/was too, without thinking. |
|  | plausible | As Beatrice ordered the necklace that was judged by the stylist on TV, Gabby did too/was too, without thinking. |
| 148 | implausible | After Charlotte was excited by the gift that friends purchased on Christmas morning, Kelly was too/did too, pretty much. |
|  | plausible | After Charlotte was excited by the gift that friends sent on Christmas morning, Kelly was too/did too, pretty much. |
| 149 | implausible | Since Paula ignored the bills that were distributed by the council every week, Lauren did too/was too, feeling guilty. |
|  | plausible | Since Paula ignored the bills that were charged by the council every week, Lauren did too/was too, feeling guilty. |
| 150 | implausible | Since Michelle was hit by the door that children broke in the lobby, Brooke was too/did too, yesterday morning. |
|  | plausible | Since Michelle was hit by the door that children pushed in the lobby, Brooke was too/did too, yesterday morning. |
| 151 | implausible | Because Will ignored the boxes that were stacked by the staff in the hallway, Tom did too/was too, walking past. |
|  | plausible | Because Will ignored the boxes that were forgotten by the staff in the hallway, Tom did too/was too, walking past. |
| 152 | implausible | Because Rob was offended by the words that hoodlums screamed last night, Kirk was too/did too, quite dramatically. |
|  | plausible | Because Rob was offended by the words that hoodlums left last night, Kirk was too/did too, quite dramatically. |
| 153 | implausible | Since Joe wanted the toy that was advertised by the TV programme daily, Jack did too/was too, really desperately. |
|  | plausible | Since Joe wanted the toy that was favoured by the TV programme daily, Jack did too/was too, really desperately. |
| 154 | implausible | Since Andrew was encouraged by the feedback that the interviewer appended to the email, Ivor was too/did too, later on. |
|  | plausible | Since Andrew was encouraged by the feedback that the interviewer referred to in the email, Ivor was too/did too, later on. |
| 155 | implausible | Because Andrea joined the gym that was reviewed by the personal trainer online, Rosie did too/was too, to exercise. |
|  | plausible | Because Andrea joined the gym that was found by the personal trainer online, Rosie did too/was too, to exercise. |
| 156 | implausible | Because Natalie was terrified by the flight that her boss booked recently, Judy was too/did too, a bit. |
|  | plausible | Because Natalie was terrified by the flight that her boss suggested recently, Judy was too/did too, a bit. |


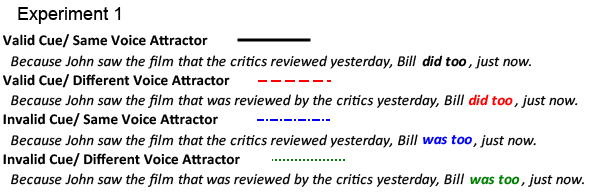

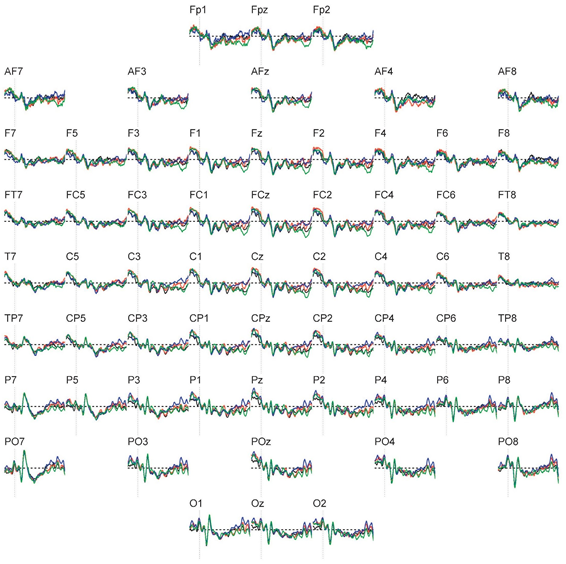


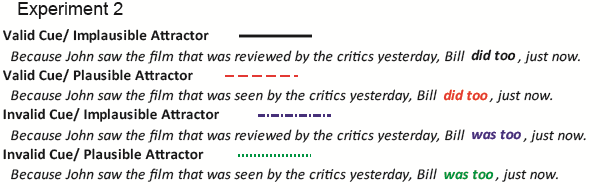


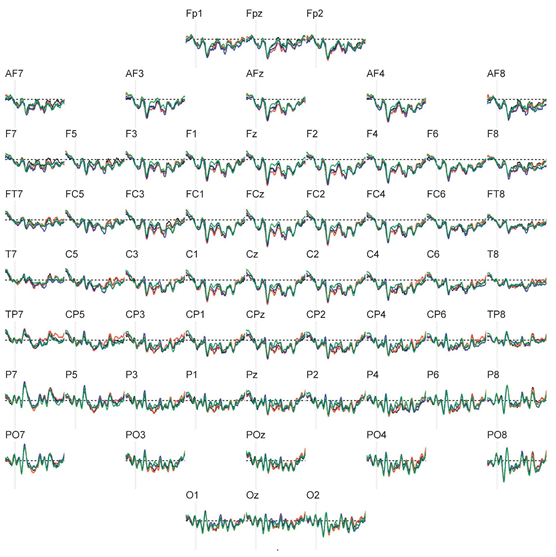

Supplement: S1 Table — (DOCX) [file pone.0206616.s001.docx]
