# Supplementary figures and images for: Cue integration during sentence comprehension: Electrophysiological evidence from ellipsis
Source: PLoS One. 2018 Nov 29;13(11):e0206616. doi: 10.1371/journal.pone.0206616 (PMC6264514; doi:10.1371/journal.pone.0206616)

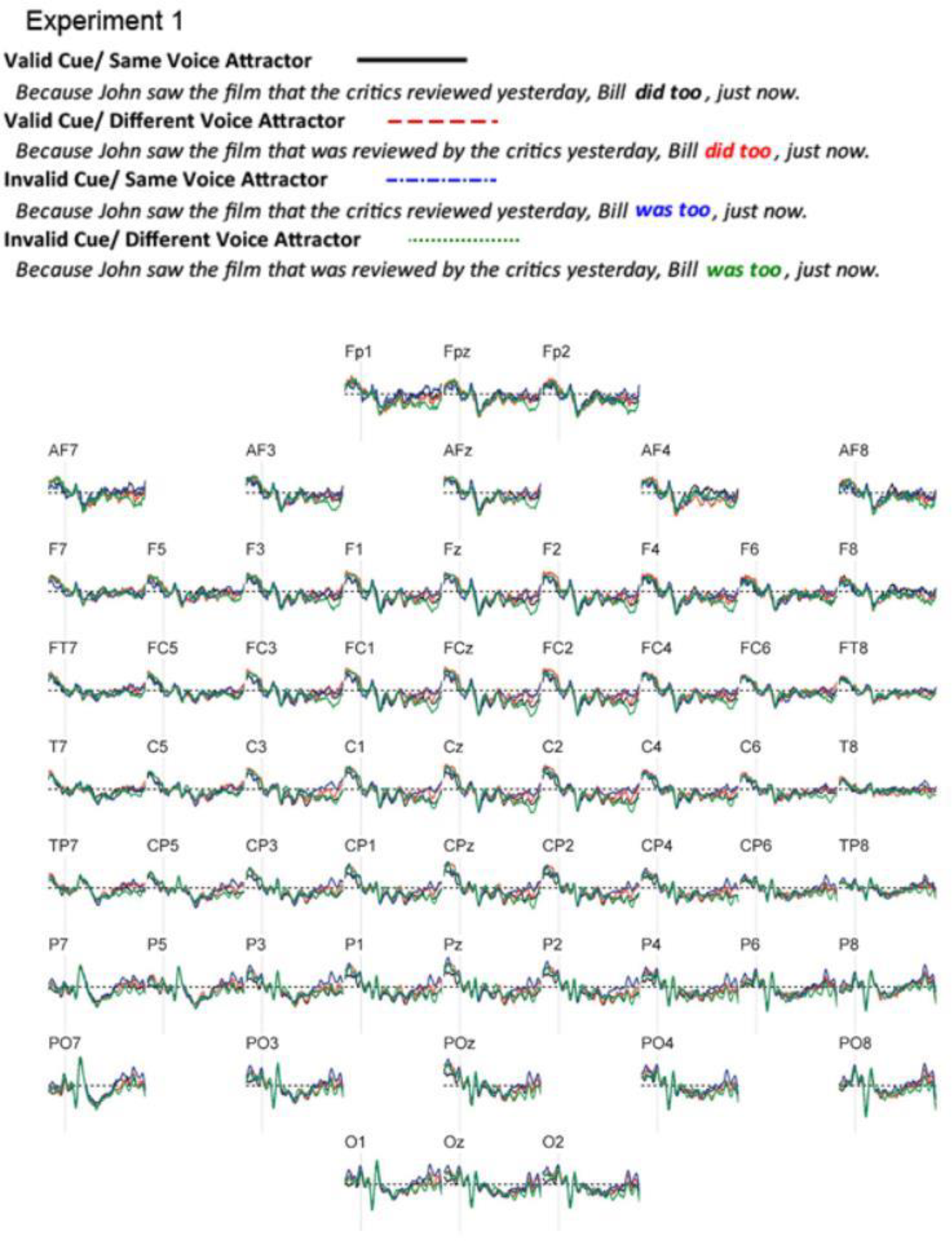

Supplement: S1 Fig — (TIF) [file pone.0206616.s002.tif]

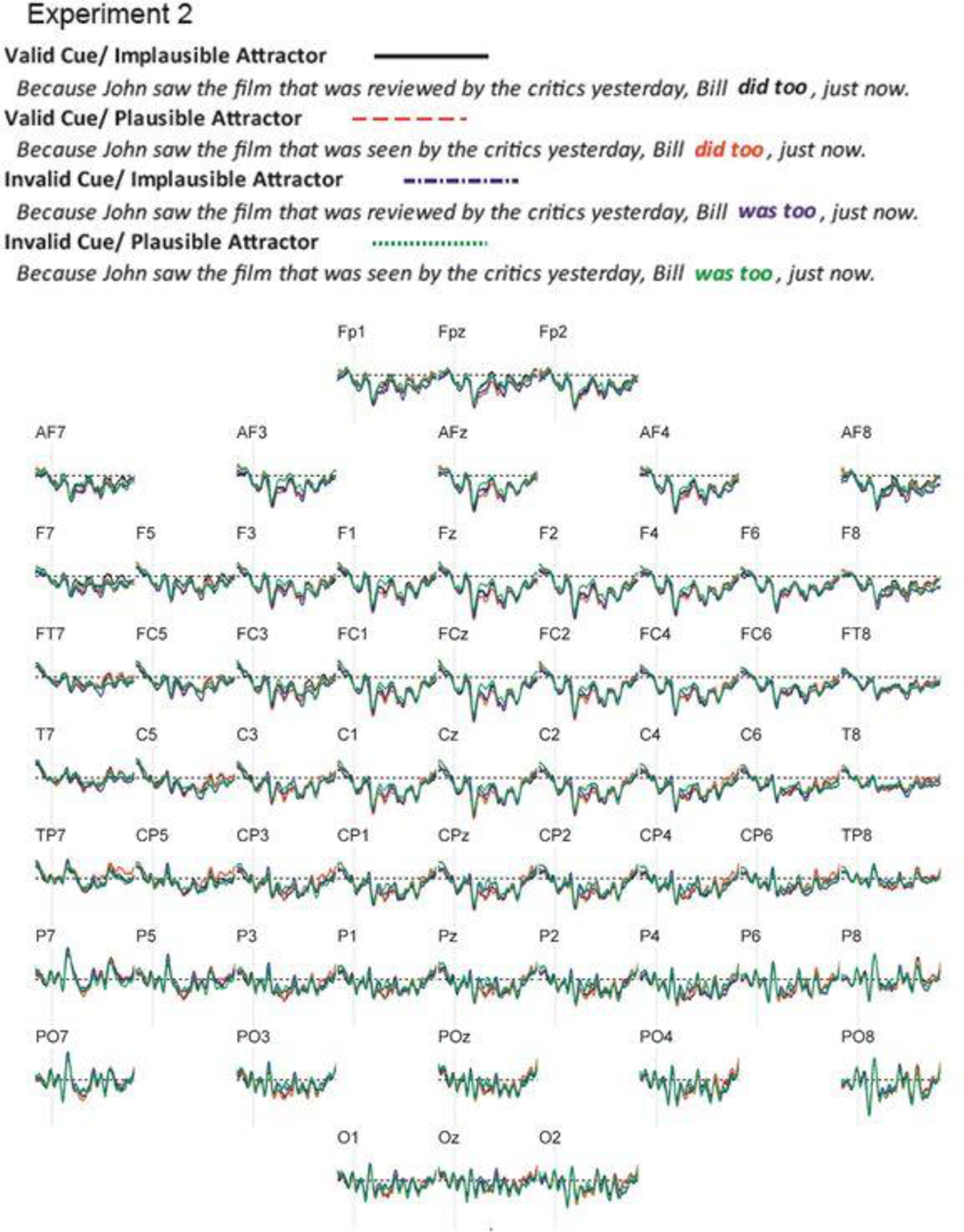

Supplement: S2 Fig — (TIF) [file pone.0206616.s003.tif]
